# Experimental constraints on a long-lived magma layer at the Martian core–mantle boundary

**DOI:** 10.1038/s41561-026-02104-z

**Published:** 2026-09-18

**Authors:** Rémy Pierru, Steeve Gréaux, Serena Dominijanni, Lianjie Man, Yoshio Kono, Sho Kakizawa, Yuji Higo, James Badro, Daniel J. Frost, Daniele Antonangeli

**Affiliations:** 1 Bayerisches Geoinstitut, Universitaet Bayreuth, Bayreuth, Germany; 2 Muséum National d′Histoire Naturelle, Sorbonne Université, UMR CNRS 7590, Institut de Minéralogie, de Physique des Matériaux et de Cosmochimie (IMPMC), Paris, France; 3 Geodynamics Research Center, Premier Institute for Advanced Studies (PIAS), Ehime University, Matsuyama, Japan; 4 CNR-ISSMC, URT Università degli studi di Roma Tre, Rome, Italy; 5 Institute of Geochemistry and Petrology, ETH Zürich, Zurich, Switzerland; 6 Department of Physics and Astronomy, Kwansei Gakuin University, Sanda, Japan; 7 Japan Synchrotron Radiation Research Institute, SPring-8, Hyogo, Japan; 8 Institut de Physique du Globe de Paris, Université Paris Cité, CNRS, Paris, France

**Keywords:** Petrology, Inner planets, Mineralogy, Geochemistry, Seismology

## Abstract

Geophysical observations from the InSight mission suggest anomalously low seismic velocities near the Martian core–mantle boundary, possibly indicating a molten silicate layer. Such a layer would have major implications for the thermal and chemical evolution of Mars, but its origin remains poorly constrained because experimental data on iron-rich Martian melts at deep-mantle conditions remain scarce. Here we use in situ ultrasonic interferometry combined with X-ray imaging and diffraction to determine the melting phase relations, ultrasonic response and chemical compositions of co-existing melts and solids under conditions relevant to the Martian core–mantle boundary. Our experiments constrain a Martian mantle solidus about 100 K lower than previous estimates. We show that incipient melting rapidly weakens the elastic response, reducing wave speeds and modifying the ultrasonic signal character. Melts generated slightly above the solidus at 18–19 GPa, equivalent to a depth of approximately 1,550 km, are strongly enriched in iron (Fe# of 0.5) and reach densities comparable to or greater than surrounding solids, implying neutral to negative buoyancy. The combination of high melt density and low wetting angles favours the formation of an interconnected dense melt layer near the core–mantle boundary from a moderately FeO-rich Martian mantle without necessarily requiring overturn of an extremely Fe-enriched primordial reservoir and remains compatible with proposed thermal models.

## Abstract (web summary)

Melts generated at the Martian core–mantle boundary would be strongly enriched in iron and have high densities favouring the formation of a molten silicate layer previously suggested from InSight seismic data, according to experimental constraints.

## Main

The InSight mission^1^ has substantially improved our knowledge of Mars’s deep interior. Seismic data from the Seismic Experiment for Interior Structure instrument have not only provided constraints on the crustal thickness and the depth of mid-mantle discontinuities^2–5^ but also revealed an unexpected low-velocity anomaly at the core–mantle boundary (CMB). The most direct interpretation of this anomaly is that it arises from an ~150-km-thick molten silicate layer above the core^6,7^. If confirmed, such a basal magma layer would play a major role in the planet’s thermal evolution, core cooling and tidal dissipation as well as the preservation of primordial geochemical reservoirs. However, its existence, physical state and origin remain uncertain. It is not yet clear under which pressure–temperature conditions deep Martian mantle material begins to melt, whether the resulting melts are dense enough to remain trapped at the base of the mantle and whether their geometry and characteristics favour retention or efficient segregation. Geodynamic simulations suggest that this low-velocity layer, rather than being a product of recent melting, may represent a long-lived remnant of a primordial magma ocean formed by segregation of dense Fe-rich residual liquids to the base of the mantle or as a result of overturn of a near-crystallized magma ocean (see, for example, refs. ^6,8,9^). However, these scenarios have been difficult to test experimentally because the phase relations, melt compositions, densities and elastic behaviour for Fe-rich Martian mantle compositions at CMB conditions remain poorly constrained. Detecting the onset of melting is itself challenging, as small melt fractions can be difficult to identify in recovered assemblages, making in situ measurements critical for resolving incipient melting at high pressure.

## Experimental strategy

**Extended Data Fig. 1:**
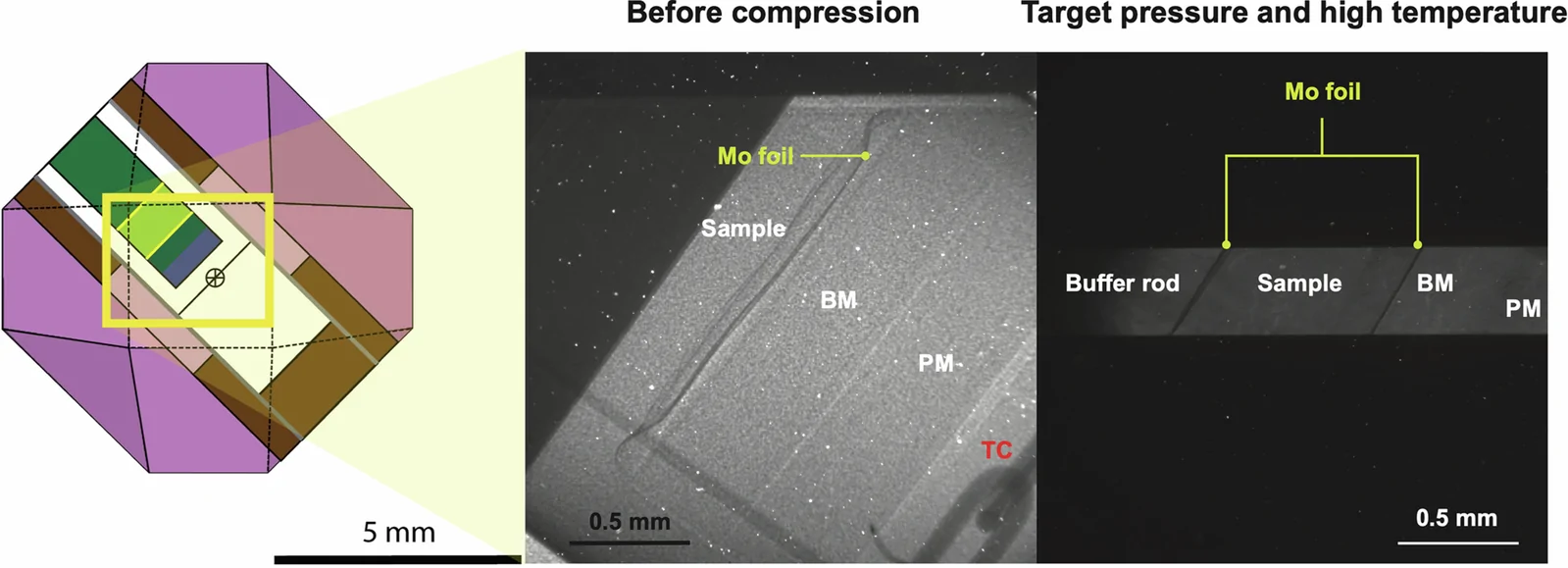
Schematic diagram of the 10/5 high-pressure cell assembly used for in situ experiments (ultrasonic and diffraction). Buffer rod, Al_2_O_3_; sample, FeO-rich martian mantle assembly previously synthesised; pressure marker (gold and hBN with MgO or NaCl); soft medium, MgO; pressure medium, (Mg,Co)O; insulator, LaCrO_3_. ⊗ denotes the thermocouple hot junction.

To investigate melting under deep Martian mantle conditions, we performed high-pressure and high-temperature experiments in a multi-anvil press, allowing in situ monitoring of the sample under conditions relevant to the Martian lowermost mantle (Extended Data Fig. 1). Using combined ultrasonic interferometry, X-ray imaging and diffraction, and ex situ textural and chemical analyses, we investigated a Martian mantle composition containing ~18 wt% FeO^10^ at pressures up to 19 GPa, corresponding to ~1,550 km depth (Supplementary Table 1).

## Detection of the onset of melting

**Fig. 1:**
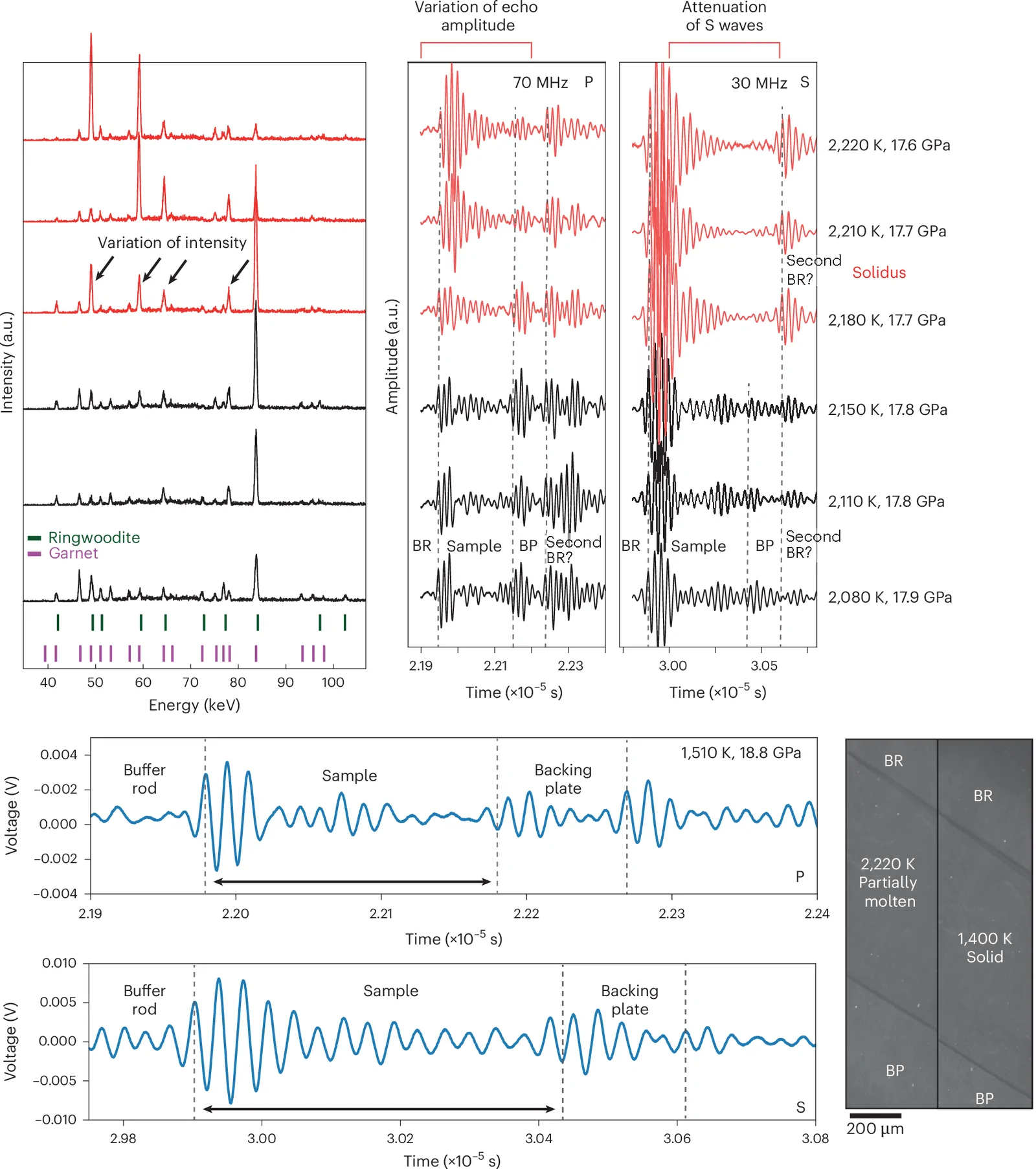
High-pressure ultrasonic interferometry and X-ray observations during melting experiments on a Martian mantle composition at approximately 18 GPa from run S3631. The upper left panel shows in situ X-ray diffraction patterns collected during heating. Tick marks indicate the main reflections of ringwoodite and garnet. The upper middle and upper right panels show time-domain ultrasonic waveforms for compressional (P) and shear (S) waves, respectively, with increasing temperature from 2,080 to 2,220 K. The sharp arrival observed at higher temperature around 3.07 × 10^−5^ s in the S wave window does not correspond to shear wave propagation through the sample but to a secondary buffer-rod-side reflection or reverberation. The opposite evolution of the backing plate (BP) and buffer rod (BR) amplitudes reflects their different acoustic paths: BR echoes are mainly sensitive to local reflection and impedance contrast at the buffer-rod side, whereas BP echoes propagate through the partially molten sample before reflection from the backing plate. This second BR signal becomes more apparent near melting, probably because strong attenuation in the partially molten sample reduces the sample and BP echoes, making buffer-rod-side reverberations more visible. It may also reflect increased back-reflection caused by melt-related impedance changes, but it was not used as an independent melting criterion. The lower left panels show representative P and S waveforms at 1,510 K and 18.8 GPa, with labelled BR, sample and BP echo regions. The lower right panel shows X-ray radiographs of the sample acquired with a high-resolution camera before and after melting. The boundaries between the BP, BR and sample are clearly marked by 2-µm molybdenum foils.

**Fig. 2:**
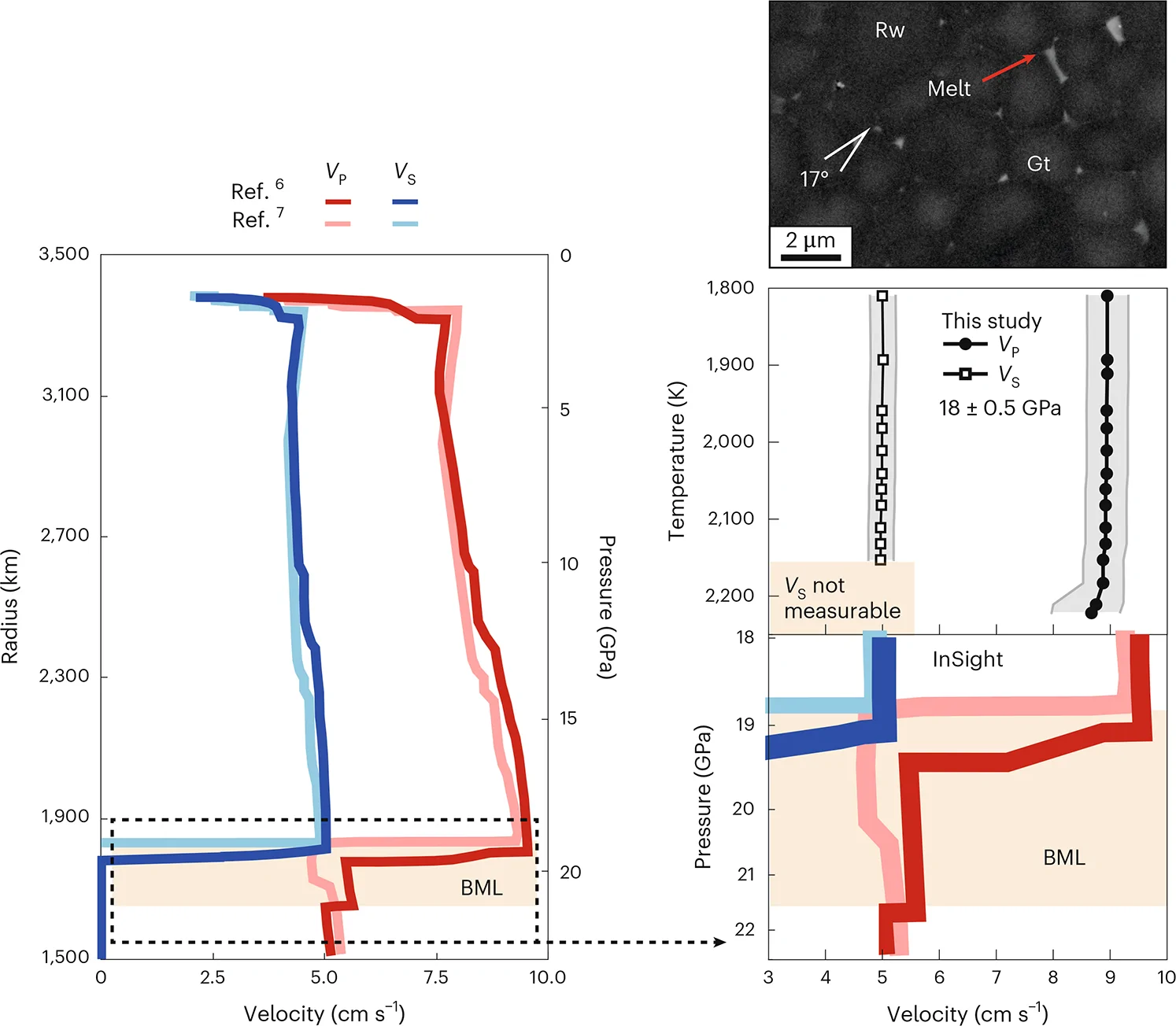
Experimental ultrasonic velocities and melt textures at Martian lowermost-mantle conditions compared with InSight-derived seismic profiles. Left: depth-dependent compressional (*V*_P_) and shear (*V*_S_) velocity profiles from recent Martian seismic models^6,7^. Right: experimental *V*_P_ and *V*_S_ measurements at 18 ± 0.5 GPa as a function of temperature. Error bars represent propagated ±1 s.d. uncertainties on ultrasonic velocities. Upon melting, *V*_P_ decreases slightly and *V*_S_ becomes no longer measurable at ultrasonic frequencies, indicating strong shear wave attenuation in the partially molten sample. The associated backscattered electron image shows interstitial Fe-rich melt along grain boundaries and triple junctions between ringwoodite (Rw) and garnet (Gt), with a low wetting angle (~20°) consistent with melt interconnection. The experimental velocity range is compared with the InSight-derived seismic profiles in the corresponding depth interval. BML, basal magma layer. Source data

**Extended Data Fig. 2:**
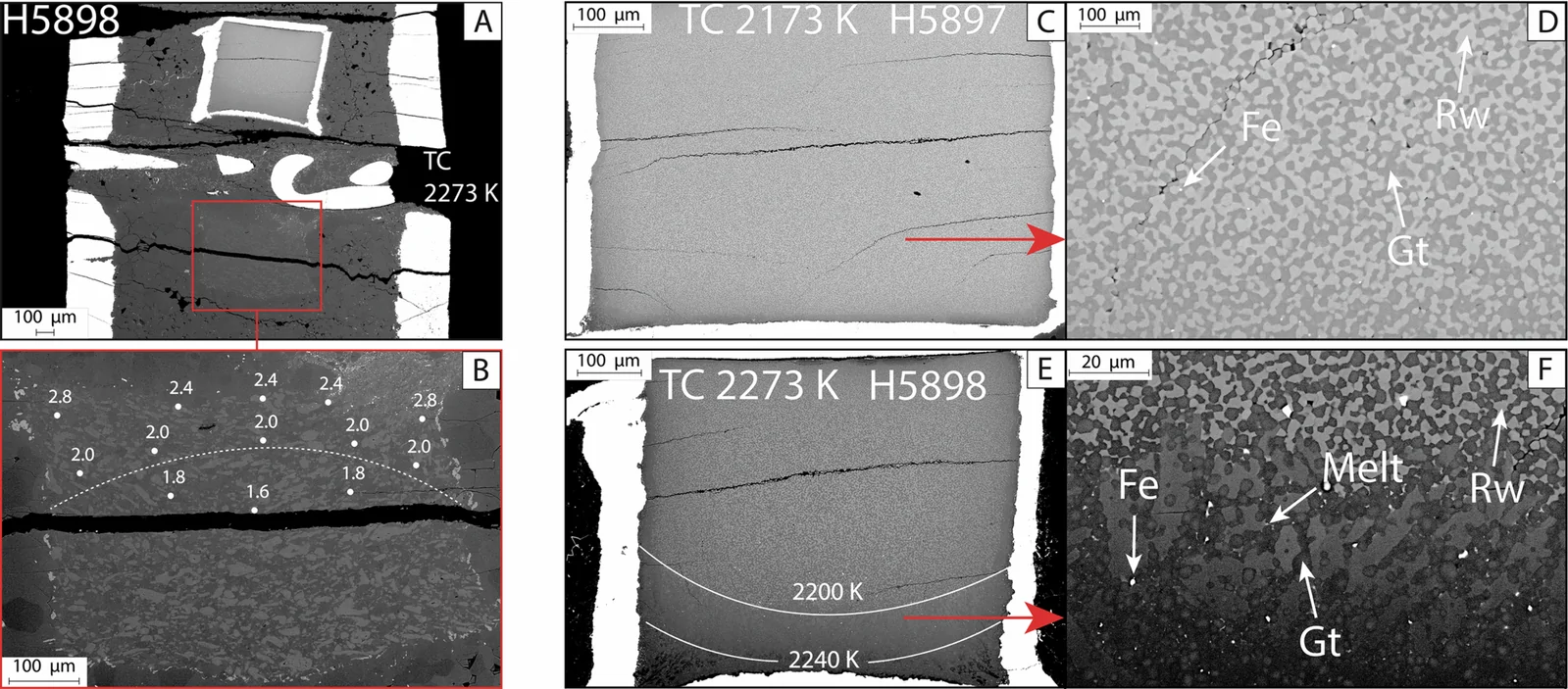
Estimation of thermal gradients, redox conditions, and melt distribution in *ex situ* quench experiments carried out at around 18 GPa. (**A**) BSE image of run H5898 showing the central portion of the BGI 10/4 assembly. The W–Re thermocouple (TC) was positioned at the center of the assembly and recorded 2273 K. A MgO–Al_2_O_3_ sample (‘periclase marker’) was placed on the opposite side of the TC to quantify thermal gradients across the assembly^15^. (**B**) Enlarged view of the periclase marker shown in (**A**). Numbers denote Al_2_O_3_ concentrations (mol%) measured in (Mg,Al)O. Because Al incorporation in periclase is temperature-dependent, these values define isotherms across the capsule. (**C**) BSE image of run H5897 quenched at a lower central temperature (2173 K). No silicate melt is observed, but exsolution of metallic Fe^0^ occurs throughout the sample, reflecting the low oxygen fugacity imposed by the molybdenum capsule. (**D**) High-magnification image of H5897 showing grains of ringwoodite (Rw), garnet (Gt), and dispersed Fe^0^. (**E**) BSE image of run H5898 (TC = 2273 K), displaying thermal gradients across the sample. Curved isotherms, inferred from Al_2_O_3_ zoning in the periclase marker, bracket the onset of melting around ~2200 ± 20 K. (**F**) Zoom of the hottest region in (**E**), showing interstitial silicate melt along grain boundaries co-existing with ringwoodite (Rw), garnet (Gt), and metallic Fe^0^. The presence of melt at 18 GPa independently confirms the solidus determined from *in situ* ultrasonic and XRD measurements, while Fe^0^ indicates redox conditions near or below the IW buffer. Source Data

The onset of melting was identified using two complementary in situ techniques: ultrasonic interferometry, which tracks changes in compressional (P) and shear (S) wave propagation (Supplementary Table 2), and X-ray diffraction, which independently records changes in phase assemblages (Supplementary Table 3). At sub-solidus conditions, both P and S waves were readily detected (Figs. 1 and 2 and Supplementary Figs. 1 and 2). Near and above the solidus, the P wave signal showed variations in amplitude and waveform shape whereas the S wave travel time either increased markedly (Supplementary Fig. 1) or could no longer be measured (Fig. 1). With increasing temperature, time-dependent variations in diffraction peak intensities indicate rapid grain rotation within the X-ray beam and the development of melt^11,12^. At 18 GPa, an increase in the P-wave attenuation was observed above the solidus, comparable to trends reported in experimental studies of terrestrial partially molten rocks^13,14^. The rapid weakening or loss of S wave reflections at low melt fractions is consistent with the strong sensitivity of shear waves to incipient melting and the development of solid–liquid interfaces along grain boundaries and triple junctions. Faint Bragg peaks progressively disappear, giving way to diffuse scattering as melting proceeds, consistent with an increasing melt fraction and enhanced melt interconnection (Supplementary Fig. 3). Complementary ex situ quench experiments at 18 GPa independently confirmed the solidus position, with temperatures constrained using the Al solubility thermometer in periclase^15^ (Extended Data Fig. 2). Our solidus temperature (*T*_s_ in Kelvin) determinations were combined with the low-pressure data of Matsukage et al.^16^ and fitted with a Simon–Glatzel parameterization $T_{\mathrm{s}}=1,400\times {\left(1+P/2.12\right)}^{1/4.97}$, where *P* is the pressure in gigapascals (*R*^2^ = 0.9968, root mean square error of 10 K) (Supplementary Table 4).

## Melt interconnection and iron enrichment of deep melts

**Fig. 3:**
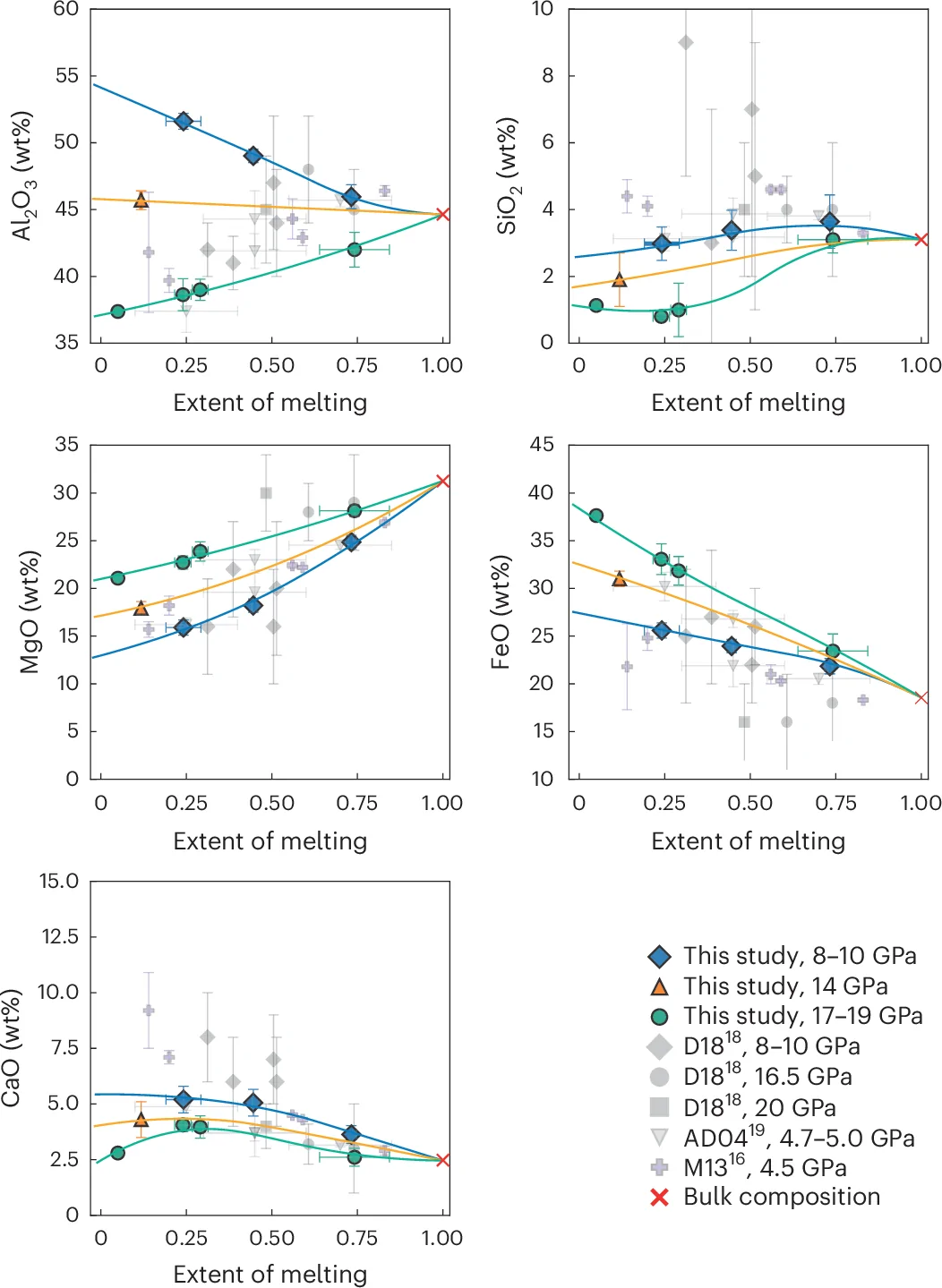
Melt compositions as a function of the extent of melting. Major element compositions of melts from this study, shown as oxide concentrations in wt.% versus the extent of melting and compared with previous Martian mantle melting experiments (M13 (ref. ^16^), D18 (ref. ^18^) and AD04 (ref. ^19^)). Symbols distinguish the different pressure ranges investigated in this study and in literature. The red cross represents the bulk starting composition at complete melting. Solid curves are guides to the eye for the compositional evolution of melts with increasing extent of melting. Vertical error bars represent ±1 s.d. analytical uncertainties calculated from multiple electron microprobe spot analyses of each phase (where *n* is the number of spot analyses; values are reported in Supplementary Table 5). Horizontal error bars represent ±1 s.d. uncertainties on melt fractions estimated from mass balance calculations. Source data

**Fig. 4:**
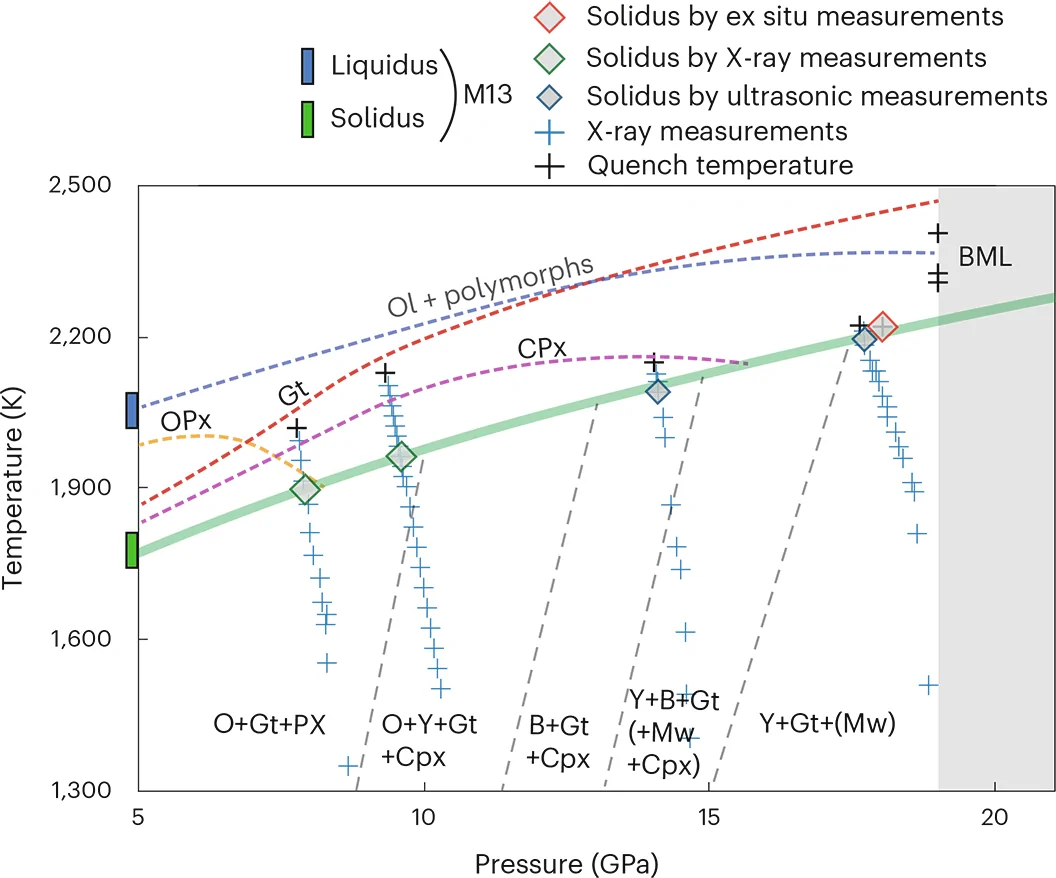
Phase relations of a Martian mantle composition as a function of pressure and temperature. Phase relations were constrained using in situ X-ray diffraction, ultrasonic measurements and ex situ characterization of quenched samples. Sub-solidus phase boundaries were traced on the basis of previously published high-pressure experimental constraints and are shown as black dashed lines to reflect their approximate nature rather than thermodynamically calculated equilibria (see, for example, refs. ^18,41,42^). The solid curve indicates the experimentally determined solidus (green). The coloured dashed lines indicate the disappearance of individual minerals from the melting assemblage, marked by phase abbreviations. Ol, olivine; PX, clinopyroxene + orthopyroxene; Gt, garnet; Y, ringwoodite; B, wadsleyite; Mw, magnesiowüstite; OPx, orthopyroxene; CPx, clinopyroxene. M13 refers to ref. ^16^. The grey-shaded region indicates the pressure range of the BML. The experimentally constrained and extrapolated stability fields of all mineral phases are consistent with the phase diagram projections at pressures below 5 GPa (ref. ^16^). Source data

**Extended Data Fig. 3:**
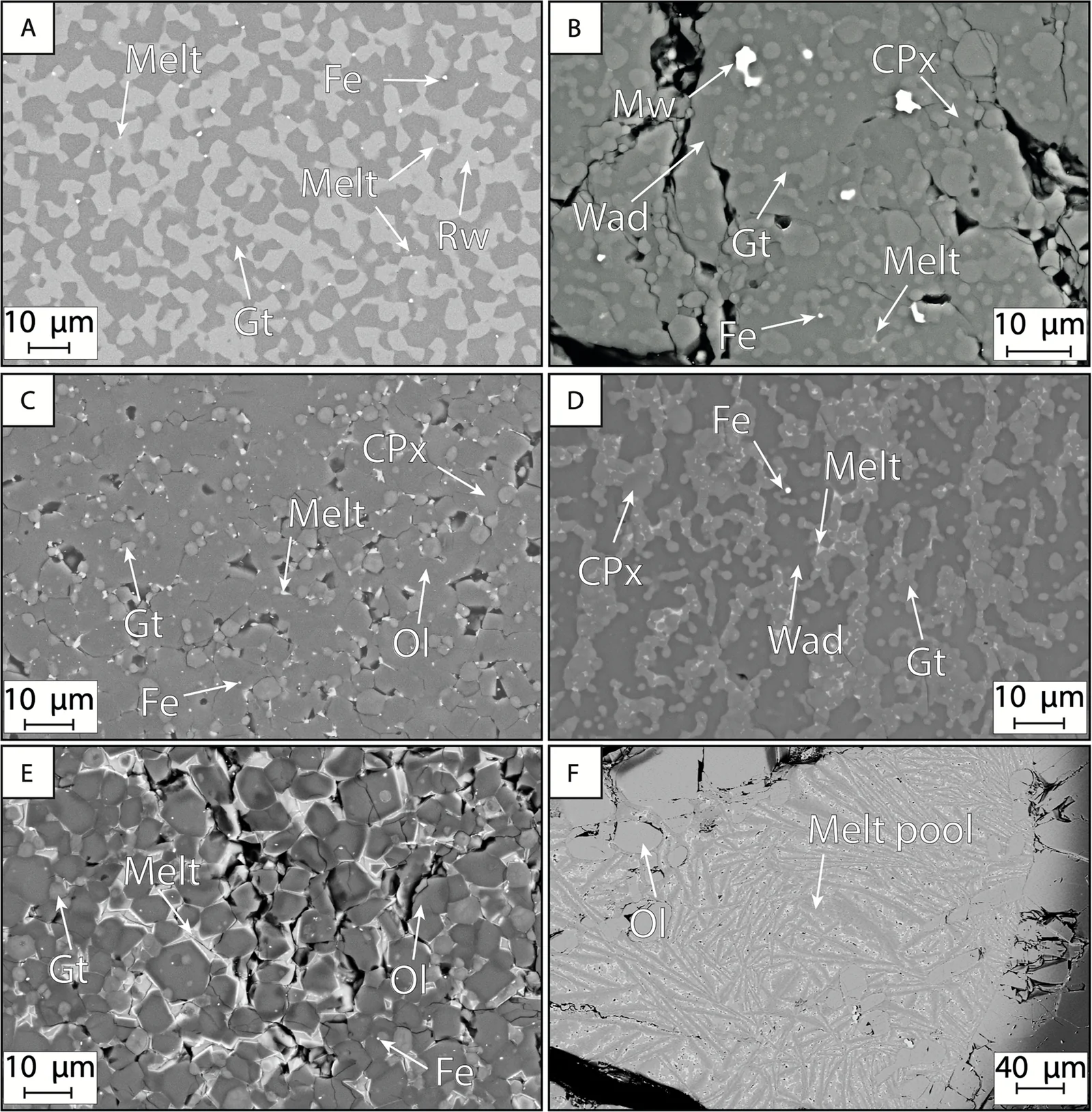
Backscattered electron (BSE) images illustrating melt fraction variations and phase distribution at different pressure-temperature conditions. Microstructural observations from BSE imaging reveal distinct textural and compositional changes associated with progressive melting: (**A**) S3631 and (**B**) S3632 (small colder region situated close to the buffer rod): Low-degree melting. Melt pockets are finely distributed along grain boundaries, suggesting incipient melting. (**C and D**) S3630 and S3632, respectively (center of the sample): increasing melt fraction leads to the formation of interconnected melt networks, indicating enhanced connectivity and a more pervasive melting regime which suggests conditions approaching the percolation threshold. (**E**) S3630 (hotspot) and (**F**) S3565: well-developed melt pathways, showing evidence of interconnected networks that facilitate melt migration. Formation of a large melt pool, displaying dendritic crystallization textures, suggesting rapid quenching from a high-temperature, largely molten state. Source Data

**Extended Data Fig. 4:**
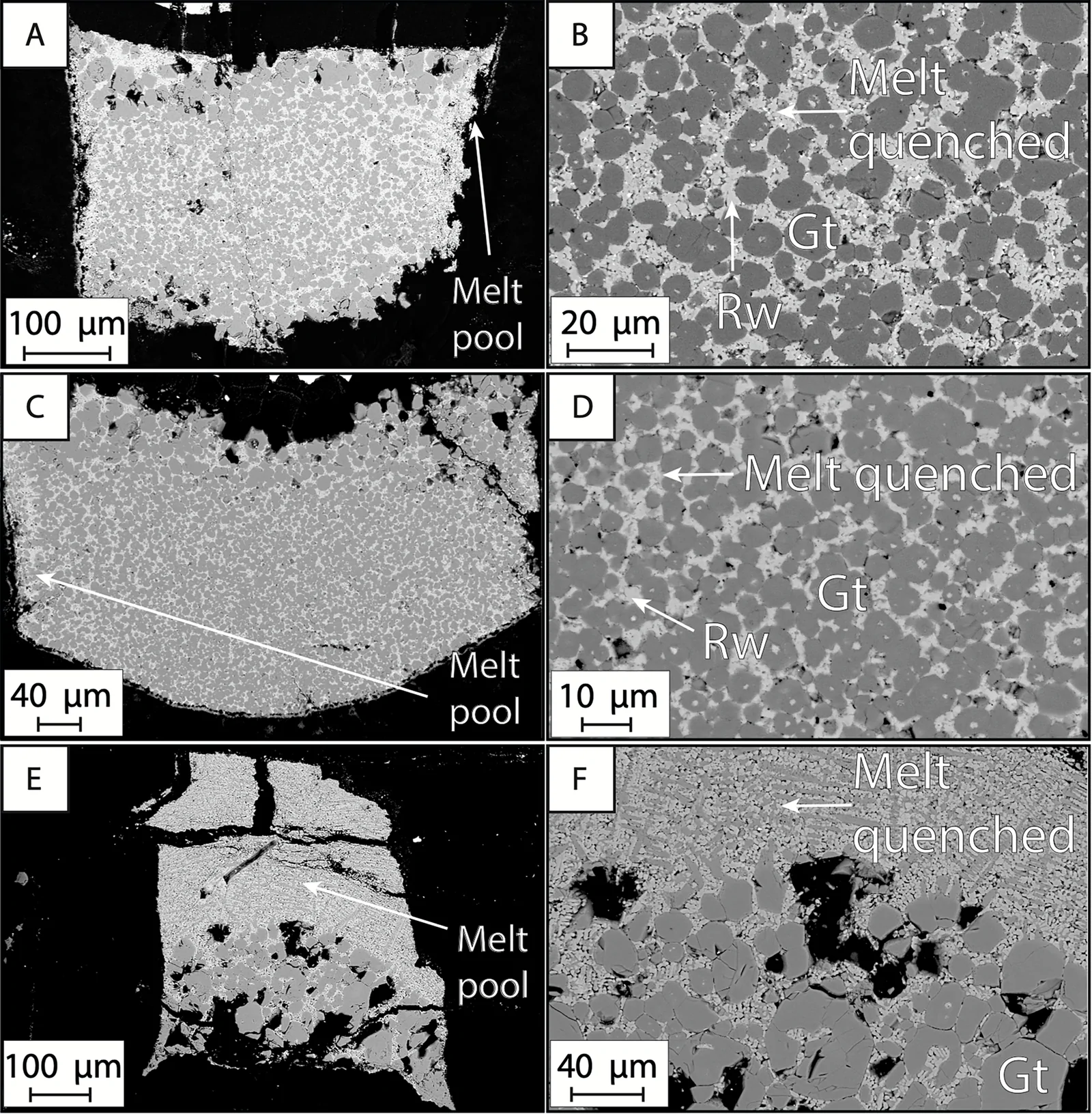
Back-scattered electron images of additional ex situ melting experiments performed at 19 GPa in graphite capsules. Panels are shown in the order S8293, S8295 and H6050. (**A–B**) Experiment S8293, showing a quenched melt pool surrounded by a fine-grained assemblage of ringwoodite (Rw), majoritic garnet (Gt) and quenched melt. (**C–D**) Experiment S8295, showing a similar high-pressure assemblage with Rw + Gt + quenched melt. (**E–F**) Experiment H6050, characterized by a much larger melt fraction and an assemblage dominated by majoritic garnet plus quenched melt. The three experiments were conducted at 19 GPa using starting powder previously equilibrated in a gas-mixing furnace at FMQ–2 to FMQ–3. Graphite capsules were used and are expected to transform to diamond at these pressure conditions. Runs were held for 10 min at target conditions before quenching. A thermocouple was placed in direct contact with the capsule for S8293 and S8295, whereas H6050 was run without a thermocouple because of the high target temperature. Source Data

**Extended Data Fig. 5:**
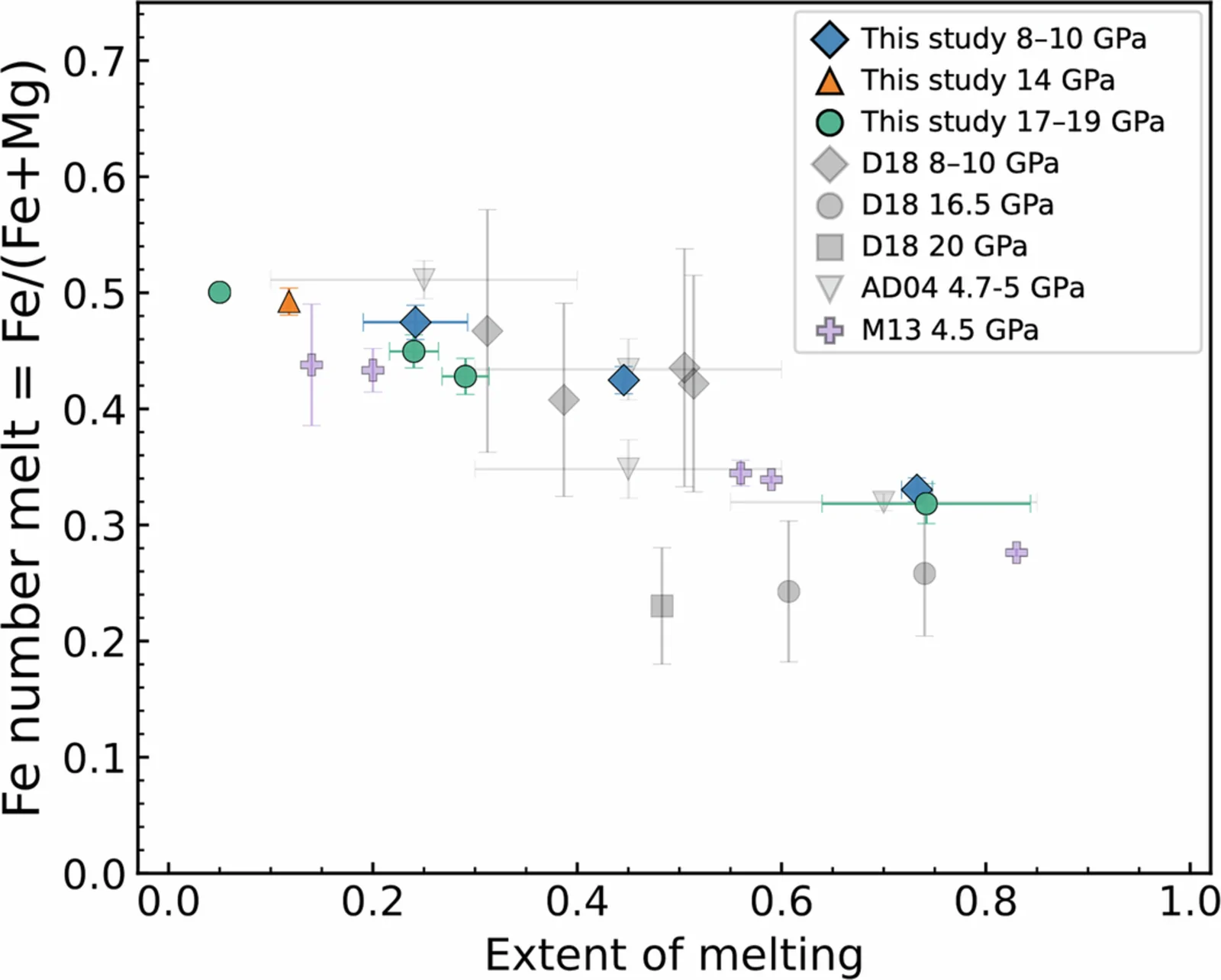
Melt Fe# as a function of melt fraction for Martian mantle melting experiments. Melt Fe# is defined as Fe/(Fe+Mg), assuming total iron as Fe. Symbols show melt compositions from this study at 8–10 GPa, 14 GPa, and 17–19 GPa, compared with previous Martian mantle melting experiments^16,18,19^. Fe# decreases with increasing melt fraction, indicating that the most Fe-rich liquids are preferentially produced during low-degree melting at high pressure. Vertical error bars represent ±1 s.d. analytical uncertainties propagated from electron microprobe analyses of FeO and MgO (n = number of spot analyses; values are reported in Supplementary Table 5). Horizontal error bars represent ±1 s.d. uncertainties on melt fractions determined by mass-balance calculations for this study. For literature data, only uncertainties explicitly reported in the original publications are shown. Matsukage et al. (2013)^16^ and Duncan et al. (2018)^18^ do not report uncertainties on mass-balance melt fractions; therefore, no horizontal error bars are shown for these data. For Agee and Draper (2004)^19^, qualitative melting categories (‘low’, ‘medium’, and ‘high’) are plotted using representative melt fractions for visualization only and do not represent measured melt-fraction uncertainties. Source Data

Recovered textures show that low melt fractions are distributed as thin films along grain boundaries and as small pockets at triple junctions (Fig. 2, Supplementary Fig. 4 and Extended Data Figs. 2 and 3). Wetting angles measured at 18 GPa remain consistently at around 20° for melt fractions estimated at ~5%, indicating that melt can form an interconnected network within the partially molten aggregate rather than remaining confined to isolated pockets (Fig. 2). At higher melt fractions, this interconnection becomes more pronounced, with the progressive development of more continuous melt-rich pathways and localized melt pools (Extended Data Figs. 3 and 4). Melt compositions show a clear pressure-dependent enrichment in FeO (Fig. 3, Supplementary Fig. 4 and Supplementary Table 5). At 8–10 GPa, low-degree melts (<10%) contain ~25–28 wt% FeO and remain relatively SiO_2_ rich (>50 wt%), consistent with early melting of pyroxene-bearing assemblages. At 14 GPa, low-degree melts contain more than 30 wt% FeO, increasing to nearly 40 wt% at 17–19 GPa. The melt Fe# $\left(\frac{\mathrm{Fe}}{\mathrm{Fe}+\mathrm{Mg}}\right)$ does not show a systematic increase with pressure at a given melt fraction; instead, the highest Fe# values occur at low melt fractions, typically below ~10% with Fe# of ~0.5 (Extended Data Fig. 5). With increasing pressure, these low-degree melts become progressively poorer in SiO_2_ and Al_2_O_3_ and richer in FeO and MgO. This pressure-dependent compositional shift reflects changes in the melting assemblage: clinopyroxene disappears from the solidus assemblage, garnet becomes more majoritic and retains much of the Ca–Al budget while Mg is increasingly hosted in wadsleyite and ringwoodite (Fig. 4 and Supplementary Figs. 5–8).

**Extended Data Fig. 6:**
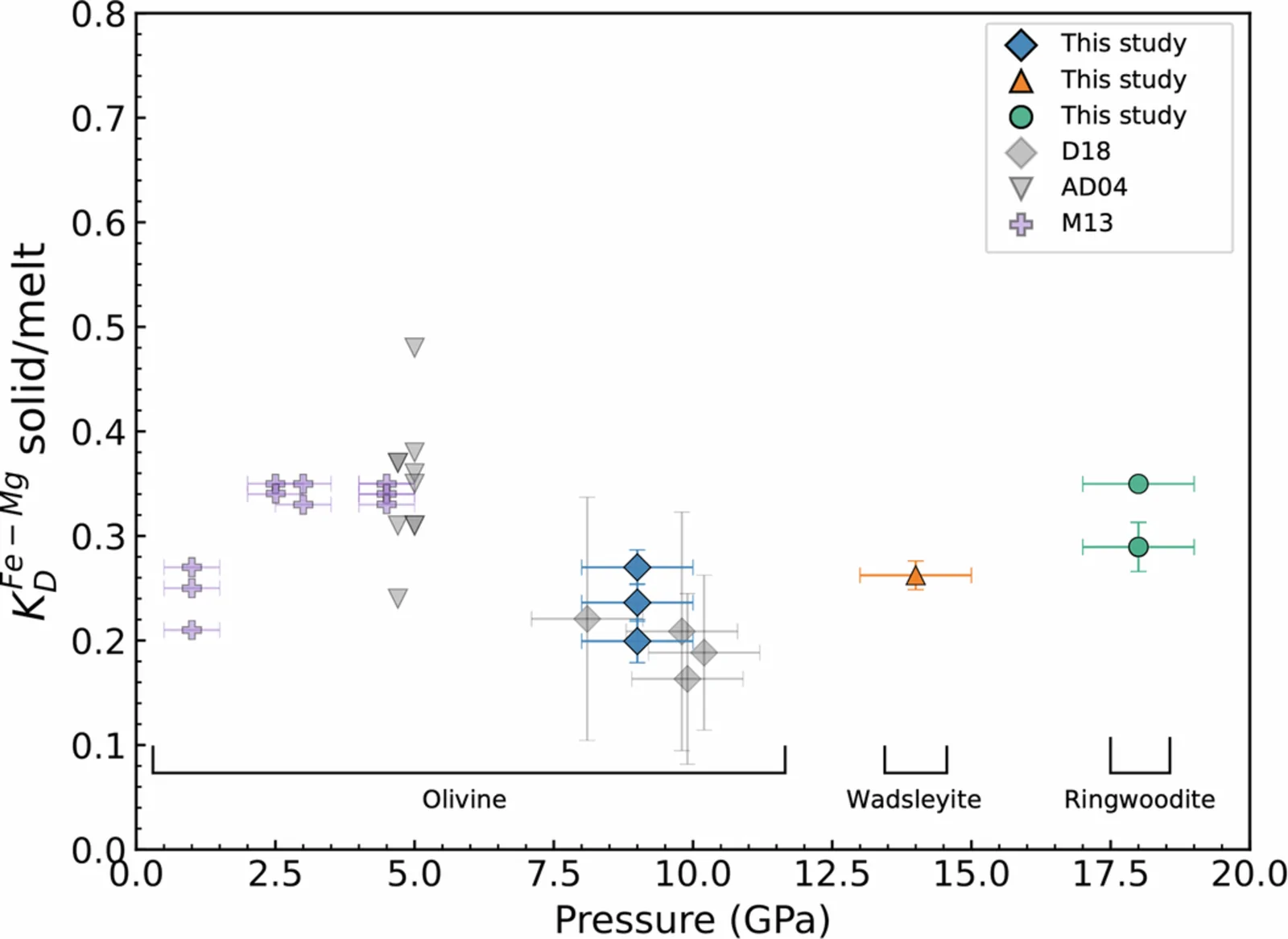
Olivine/ringwoodite–melt Fe–Mg exchange coefficient as a function of pressure. ${\rm{K}}_{\rm{D}}^{{\rm{Fe}}-{\rm{Mg}}}$ is defined as ${\left({\rm{FeO}}/{\rm{MgO}}\right)}_{{ol}}/{\left({\rm{FeO}}/{\rm{MgO}}\right)}_{\rm{melt}}$. Symbols show values from this study at 8–10 GPa, 14 GPa, and 17–19 GPa, compared with published Martian mantle melting experiments^16,18,19^. The values from this study overlap with previous high-pressure constraints and remain close to the canonical Martian olivine–melt equilibrium range at low melt fractions (0.35)^17^. Vertical error bars represent ±1 s.d. uncertainties propagated from electron microprobe analyses of olivine and melt compositions (n = number of spot analyses; values are reported in Supplementary Table 5). Horizontal error bars represent pressure uncertainties for this study and, where explicitly reported, for literature data. Source Data

**Extended Data Fig. 7:**
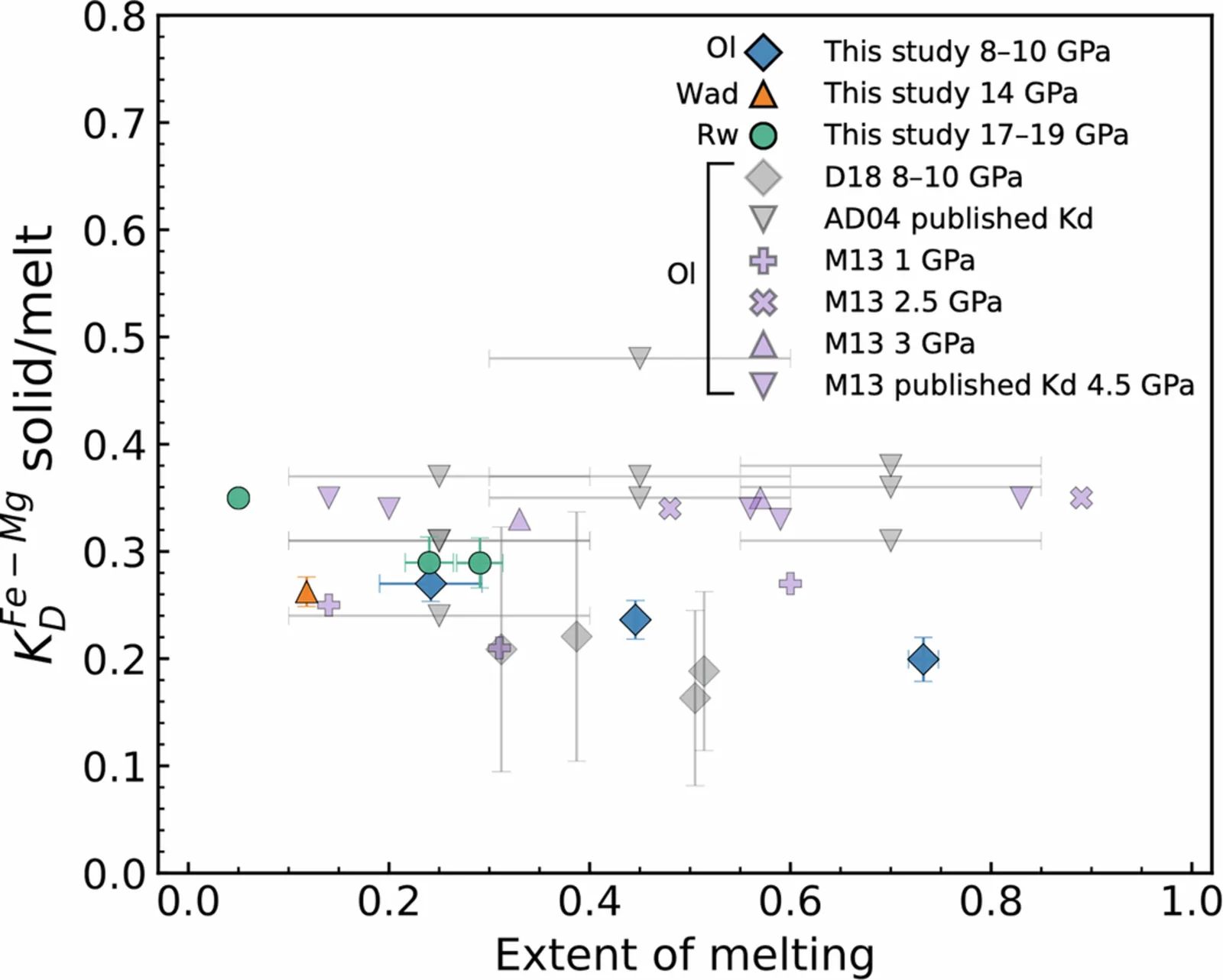
Olivine/ringwoodite–melt Fe–Mg exchange coefficient as a function of melt fraction. ${\rm{K}}_{\rm{D}}^{{\rm{Fe}}-{\rm{Mg}}}$ is defined as ${\left({\rm{FeO}}/{\rm{MgO}}\right)}_{\rm{ol}}/{\left({\rm{FeO}}/{\rm{MgO}}\right)}_{\rm{melt}}$. Symbols show values from this study at 8–10 GPa, 14 GPa, and 17–19 GPa, compared with published Martian mantle melting experiments^16,18,19^. In the present study, ${\rm{K}}_{\rm{D}}^{{\rm{Fe}}-{\rm{Mg}}}$ varies more clearly with melt fraction than with pressure, decreasing from values close to ~0.35 at low melt fraction to ~0.20 at high melt fraction in the 17–19 GPa experiments. Vertical error bars represent ±1 s.d. uncertainties propagated from electron microprobe analyses of olivine and melt compositions (n = number of spot analyses; values are reported in Supplementary Table 5). Horizontal error bars represent ±1 s.d. uncertainties on melt fractions determined by mass-balance calculations for this study. For literature data, only uncertainties explicitly reported in the original publications are shown. Matsukage et al. (2013)^16^ and Duncan et al. (2018)^18^ do not report uncertainties on mass-balance melt fractions; therefore, no horizontal error bars are shown for these data. For Agee and Draper (2004)^19^, qualitative melting categories (‘low’, ‘medium’, and ‘high’) are plotted using representative melt fractions for visualization only and do not represent measured melt-fraction uncertainties. Source Data

The olivine polymorph–melt Fe–Mg exchange coefficient ${K}_{{\rm{D}}}^{{\mathrm{Fe}}\mbox{-}{\mathrm{Mg}}}=\frac{{\left({\mathrm{FeO}}/{\mathrm{MgO}}\right)}_{\mathrm{ol}}}{{\left({\mathrm{FeO}}/{\mathrm{MgO}}\right)}_{\mathrm{melt}}}$, provides another constraint on the origin of the Fe-rich liquids. The measured ${K}_{{\rm{D}}}^{{\mathrm{Fe}}\mbox{--}{\mathrm{Mg}}}$ values remain broadly consistent with previous high-pressure Martian mantle experiments and with expected values for silicate–melt equilibrium^16–19^ (Extended Data Figs. 6 and 7). Although the olivine–melt ${K}_{{\rm{D}}}^{\mathrm{Fe}\mbox{--}\mathrm{Mg}}$ tends to decrease with pressure above ~8 GPa, this trend can be modified, or even partly reversed, upon transformation to high-pressure olivine polymorphs such as ringwoodite^20^, which may explain the limited variation in ${K}_{{\rm{D}}}^{\mathrm{Fe}\mbox{--}\mathrm{Mg}}$ across our experiments. With increasing pressure, low-degree melts become progressively poorer in SiO_2_ at high pressure, below 40 wt%, consistent with high-pressure melting relations in simple silicate systems^21^. Lower SiO_2_ contents increase the relative proportions of MgO and FeO and affect the Fe–Mg exchange, because ${K}_{{\rm{D}}}^{\mathrm{Fe}\mbox{--}\mathrm{Mg}}$ is sensitive to the melt composition, particularly the SiO_2_ content^22^. The early involvement of ringwoodite and the relative incompatibility of Fe favour Fe enrichment of the liquid, leaving more Mg- and Al-rich residual solids. The associated increase in FeO and MgO and decrease in SiO_2_ and Al_2_O_3_ raise the melt density, while depolymerization may also affect the viscosity and promote melt mobility, downward segregation and accumulation at depth.

## Melt–solid density contrast near the Martian CMB

**Fig. 5:**
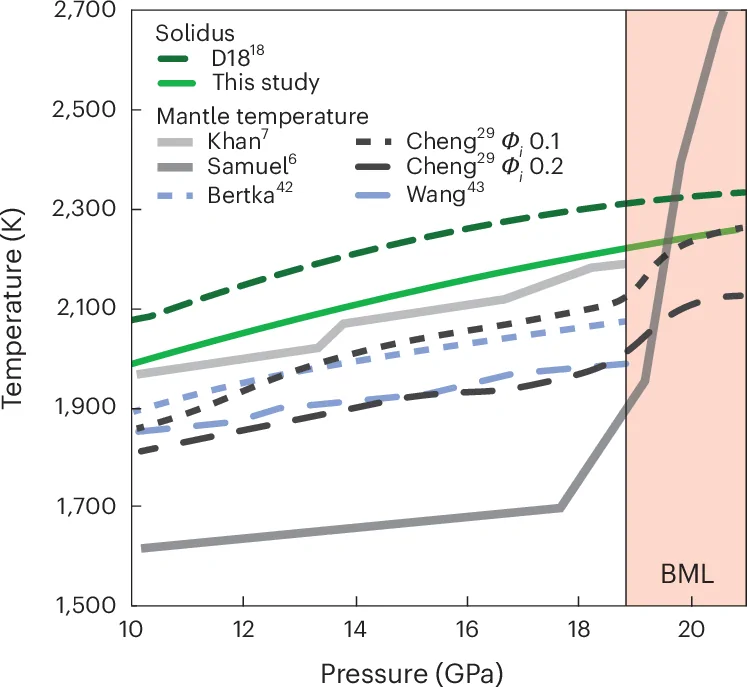
Martian mantle solidi and lowermost-mantle thermal profiles. The solid green curve represents the solidus determined in this study, whereas the dashed dark-green curve shows the previous experimental solidus by Duncan et al.^18^. Grey and blue curves indicate mantle temperature profiles from published Martian interior and thermal evolution models, including refs. ^6,7,29,42,43^, as indicated in the legend. The Khan et al.^7^ profile corresponds to the mean of 1,000 interior models. For Cheng et al.^29^, the two curves correspond to models with different initial interstitial porosity, *ϕ*_i_ = 0.1 and *ϕ*_i_ = 0.2, which control the partitioning of heat-producing elements between the basal magma ocean and the overlying mantle. The pink-shaded region marks the pressure range of the proposed BML. Across these different models, temperatures near the Martian core–mantle boundary are expected to lie broadly between ~1,900 and 2,200 K. The revised solidus obtained in this study reduces the temperature gap between proposed lowermost-mantle temperatures and the onset of melting near the core–mantle boundary. Source data

**Extended Data Fig. 8:**
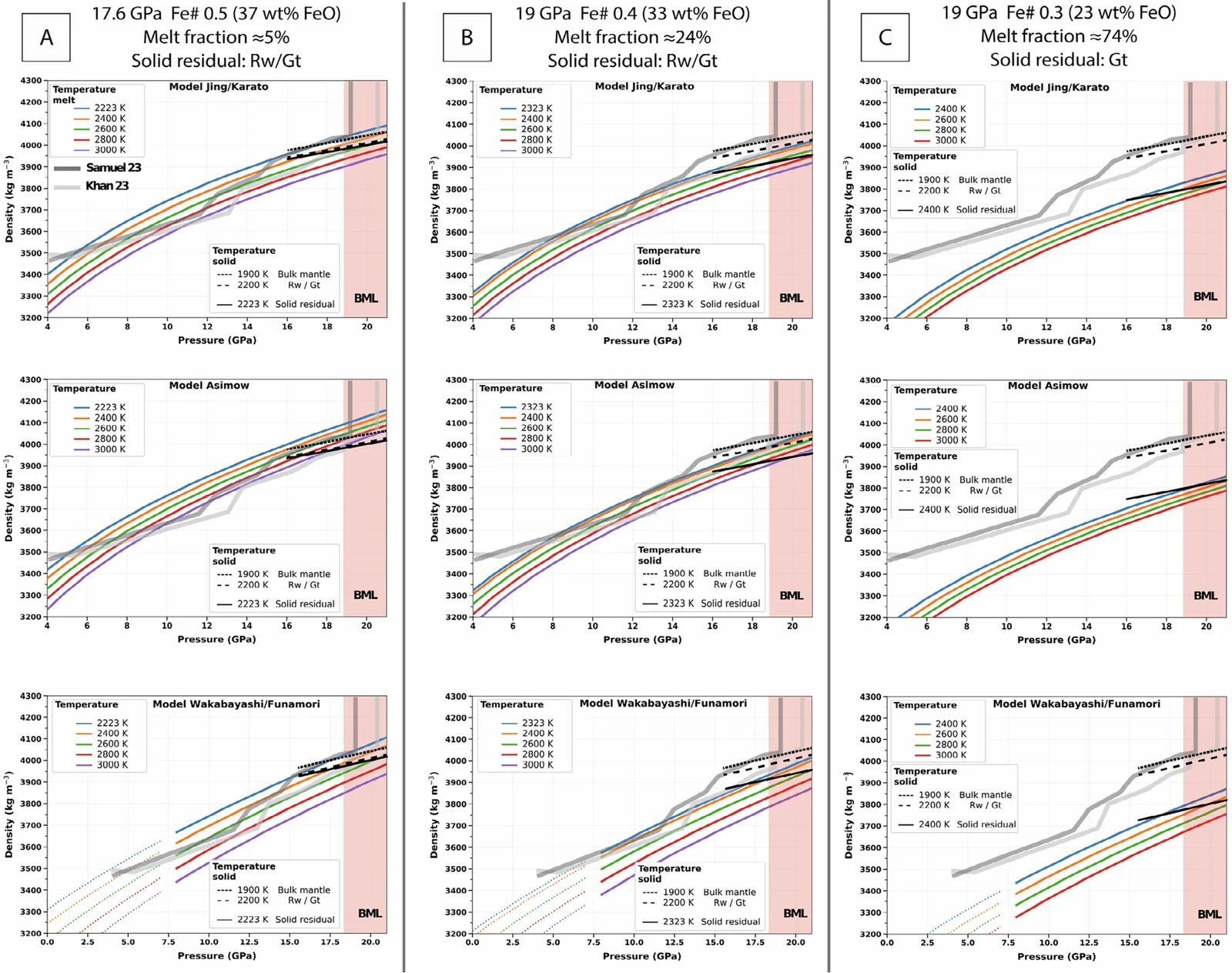
Calculated melt-density profiles for samples S3631 (A), S8295 (B), and H6050 (C). These three samples from 17–19 GPa represent progressively increasing melt fractions, from ~5% in S3631 to ~24% in S8295 and ~74% in H6050, accompanied by a decrease in FeO content from 37 to 33 and 23 wt.%, respectively. Liquid densities were independently calculated using different equations of state to assess the sensitivity of the results to the choice of liquid EoS^23–25^. Solid-density profiles were calculated with thermoelastic modelling both for the residual assemblage in equilibrium with each experimentally produced melt and for the undepleted bulk ringwoodite–garnet mantle derived from the MA17 composition reported in Supplementary Table 5. The thickness of the coloured band represents the corresponding 1σ uncertainty in the calculated garnet–ringwoodite aggregate density, obtained by Monte Carlo propagation of the thermoelastic parameter uncertainties. In each panel, the blue curve corresponds to the temperature at which the melt was produced experimentally, whereas the other colored curves illustrate the effect of higher temperatures on liquid density. As expected, increasing temperature lowers melt density, while increasing melt fraction is associated with lower FeO contents and therefore systematically lower densities from A to C. The pink shaded field marks the proposed pressure range of the Martian basal magma layer and the gray curves show the density estimates of Samuel et al.^6^ and Khan et al.^7^. The propagated 1σ relative uncertainties over the BML pressure range are approximately 2% for Asimow ^23^ and Wakabayashi–Funamori models^24^, and 4 % for the Jing–Karato model^25^ (Supplementary Table 6). These values were obtained by Monte Carlo propagation of published EoS parameter uncertainties and likely represent conservative, potentially overestimated bounds because parameter covariance matrices were unavailable and the parameters were therefore sampled independently. Source Data

To assess melt buoyancy, we compared the densities of liquids and co-existing solid assemblages over a range of melt fractions representative of our experiments at 17–19 GPa (Extended Data Fig. 8). Liquid densities were calculated along different isotherms from the measured melt compositions using three independent high-pressure equations of state (EoS) for silicate liquids^23–25^. Solid densities were calculated from thermoelastic modelling both for the residual assemblage in equilibrium with each melt and for the corresponding bulk ringwoodite–garnet mantle composition between 16 and 21 GPa (Extended Data Fig. 8 and Supplementary Table 5). Because CMB mantle temperatures remain uncertain, bulk mantle densities were calculated over a plausible range of 1,900–2,200 K (Fig. 5). At low melt fraction (~5%), the melt is strongly enriched in FeO and, at its experimental production temperature (2,223 K), reaches densities comparable to or greater than both the co-existing residual assemblage and the bulk mantle at pressures between 19 and 21 GPa. This indicates neutral to negative buoyancy, favouring downward segregation and accumulation near the CMB. This dense-melt behaviour may persist up to approximately 2,400–2,800 K, depending on the liquid EoS. At intermediate to high degrees of melting (~24–74%), the melts are poorer in FeO and less dense than the bulk mantle between 19 and 21 GPa, although they remain denser than their co-existing residual assemblages at their experimental production temperatures (2,323 K and 2,400 K, respectively). As the temperature increases, the melt-residue density contrast progressively reduces and eventually reverses. These results show that gravitational stability is more robust for low-degree, Fe-rich melts. As melting proceeds, progressive Fe depletion reduces the melt density, making higher-degree melts increasingly buoyant and less likely to form or sustain a dense basal silicate layer.

## Ultrasonic signatures of incipient melting

Our ultrasonic measurements, besides pinning the solidus temperature, capture the first-order effects of incipient melting on wave propagation in Fe-rich Martian mantle assemblages. Below the solidus, both P and S waves are readily detected, with corresponding velocities of *V*_P_ ≈ 9 km s^−1^ and *V*_S_ ≈ 5 km s^−1^, broadly consistent with InSight-derived lower-mantle profiles (Fig. 2, Supplementary Fig. 2 and Supplementary Table 2).

Upon melting, both velocities decrease, with the effect larger for *V*_S_, which is highly sensitive to melt films, isolated melt pockets and solid–liquid interfaces. At 14 GPa, both *V*_P_ and *V*_S_ remain measurable across the onset of melting (Supplementary Fig. 1), although these measurements carry larger propagated uncertainties. The reduction in *V*_S_ is substantial, reaching ~18% just above the solidus and ~33% at higher melt fraction. By contrast, the apparent reduction in *V*_P_ is smaller, up to ~4%, and partly overlaps with the propagated uncertainties, indicating that the weak effect of low melt fractions on *V*_P_ may remain difficult to resolve within the present experimental precision. At 18 GPa, *V*_P_ shows only a modest decrease of ~1% at low melt fraction, again comparable to the propagated uncertainty envelope, whereas *V*_S_ becomes no longer measurable at ultrasonic frequencies (Fig. 2). Low dihedral angles can reduce grain–grain contiguity and lower effective elastic moduli, making *V*_S_ particularly sensitive to small amounts of well-connected melt^26^. Beyond this geometric effect, motion at solid–melt interfaces may further enhance the attenuation and velocity reduction through pressure-induced boundary motion^27^.

These effects are especially important in partially molten assemblages, where the melt distribution, thermal gradients, evolving acoustic path length and anelastic relaxation can affect the measured velocities through frequency-dependent dispersion and attenuation^28^. Nevertheless, although ultrasonic measurements at tens of megahertz cannot be directly extrapolated to seismic frequencies, this comparison remains instructive.

Our experiments clearly show that small amounts of interconnected melt generate attenuation and reduce ultrasonic velocities. However, the observed reductions appear largely insufficient to reproduce the large low-velocity anomalies inferred near the Martian CMB. This suggests the presence of extended melt-rich regions with much higher melt fractions rather than pervasive low-degree melting.

## A revised Martian solidus

Our measurements place the solidus of a Martian mantle composition approximately 100 K lower than earlier estimates (Fig. 5). This difference cannot be attributed to bulk composition or oxygen fugacity; rather, it reflects the improved sensitivity of our in situ experimental approach. The observation of metallic Fe^0^ in the recovered experiments indicates oxygen fugacities near or below the iron–wüstite (IW) buffer, consistent with reducing conditions expected close to the Martian CMB. Unlike the previous high-pressure solidus based on quenched textures^18^, our combined ultrasonic and X-ray approach detects incipient melting in situ, probing melt fractions below conventional ex situ detection limits. This lower solidus directly affects the thermal feasibility of basal melting, although the temperature of the Martian lowermost mantle remains uncertain across published thermal models (Fig. 5). For warm models such as those of Samuel et al.^6^, basal temperatures exceed any reasonable Martian mantle solidus, making melting readily achievable, whereas precise solidus determination is more critical for cooler profiles, such as those of Cheng et al.^29^. In the latter case, the Duncan et al.^18^ solidus remains too high relative to the predicted basal mantle temperatures to allow melting. By contrast, our revised, lower solidus is compatible with melting under moderate heat-producing element enrichment or slightly warmer basal conditions. If the lowermost mantle reaches temperatures above the solidus, even small melt fractions can effectively segregate and accumulate above the core, leading to the formation of a basal magma layer without requiring exceptionally high temperatures, high enrichments in Fe or high enrichment in heat-producing elements, even though these factors could further enhance melt production.

## Gravitational stability and accumulation of Fe-rich basal melts

**Fig. 6:**
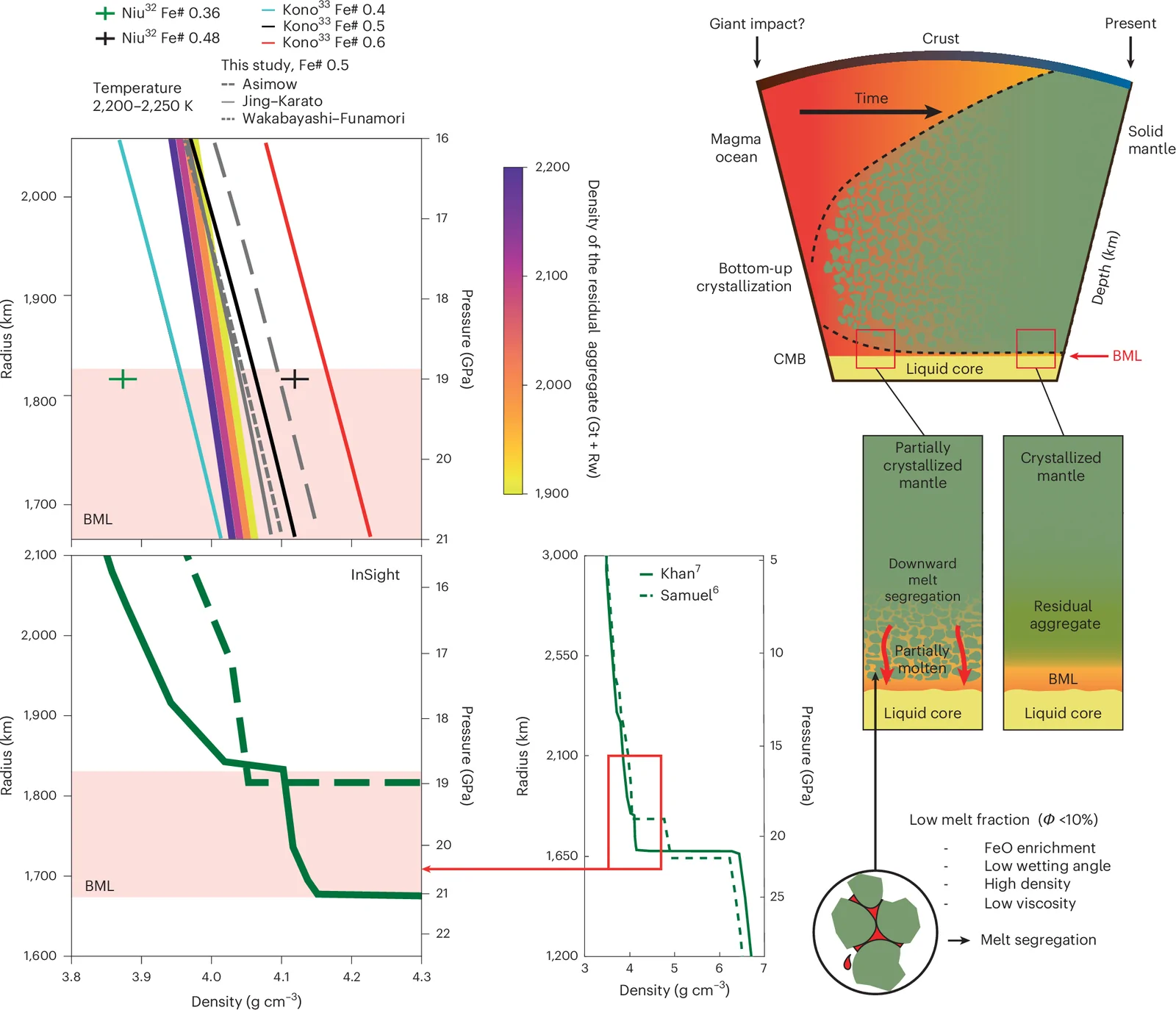
Gravitational separation of FeO-rich melts and development of a basal silicate melt layer at the Martian core–mantle boundary. Left: the calculated density of an Fe-rich silicate melt (Fe# of ~0.5; S3631) produced in this study, compared with the density of the undepleted bulk mantle, represented by a ringwoodite–garnet aggregate derived from the MA17 composition reported in Supplementary Table 5. Solid-density profiles were calculated over a plausible range of lowermost mantle temperatures from 1,900 to 2,200 K and are shown as coloured bands. The corresponding density profiles of the Martian lowermost mantle inferred from InSight-based models^6,7^ are also shown for comparison. The thickness of the coloured band represents the corresponding 1*σ* uncertainty in the calculated garnet–ringwoodite aggregate density, obtained by Monte Carlo propagation of the thermoelastic parameter uncertainties. The figure also includes experimentally determined melt densities^33^ and molecular dynamics calculations^32^. The propagated 1*σ* relative uncertainties over the BML pressure range are approximately 2% for Asimow and Wakabayashi–Funamori models, and 4% for the Jing–Karato model (Supplementary Table 6). These values were obtained by Monte Carlo propagation of published equation-of-state parameter uncertainties and probably represent conservative, potentially overestimated bounds because parameter covariance matrices were unavailable and the parameters were therefore sampled independently. Right: a conceptual model for the generation, segregation and preservation of a basal magma layer. Low-degree melting near the CMB, or residual melt produced during magma ocean crystallization, generates FeO-rich liquids with low melt fractions ($\varPhi < 10\%$), low wetting angles (~20°) and high densities. These properties favour melt interconnection, downward percolation and pooling at the base of the mantle, potentially forming a dense, melt-rich silicate layer above the liquid core. Source data

Our experiments provide a plausible mineral-physics framework for the proposed BML, although the relevant InSight observations remain open to alternative interpretations, including mantle scattering that does not uniquely require a molten or partially molten layer^6,7,30,31^. Very low-degree melts (<5%) with low dihedral angles produced within the pressure and depth interval corresponding to the proposed BML become both Fe rich and sufficiently dense to be neutrally to negatively buoyant relative to the surrounding bulk mantle (Fig. 6 and Supplementary Table 6). For representative melts with Fe# of ~0.5, densities calculated along a reference melt temperature profile (Supplementary Fig. 9) reach ~4.1 g cm^−3^ near 18–20 GPa, overlapping with the density range inferred for dense basal silicate layers in some InSight-based models, although this comparison remains approximate owing to uncertainties in seismic inversions. This is consistent with recent molecular dynamics and experimental constraints suggesting that stable Martian basal melts require >30 wt% FeO and densities near ~4.1 g cm^−3^ (refs. ^32,33^). Whereas these previous studies set a lower boundary for the physical conditions required for stable dense basal melts, our experiments directly show that such Fe-rich, dense liquids can indeed be produced by low-degree melting of a Martian mantle composition at CMB pressures.

The density range proposed by Khan et al.^7^ for a basal magma layer of ~4.0–4.1 g cm^−3^ is consistent with our calculated Fe-rich liquids and could reflect low-degree melts accumulated at the CMB. In contrast, the higher densities inferred by Samuel et al.^6^, near ~4.8 g cm^−3^, would require even more Fe-rich liquids, additional dense components or compositional stratification. Liquids with Fe# >0.5–0.6, potentially produced by fractional crystallization or progressive melt–solid segregation, could provide a pathway towards denser basal compositions (Fig. 6).

## Compositional limits on basal melt stability

If the starting mantle composition were less FeO rich than that investigated here, low-degree melts would have lower Fe# and lower densities, weakening their tendency to accumulate at the CMB. For example, assuming similar Fe–Mg partitioning and a Yoshizaki–McDonough bulk-Mars composition^34^, featuring ~15 wt% FeO instead of ~18 wt%, would produce melts with Fe# of ~0.40–0.43 at 18 GPa, compared with Fe# of 0.5 in our melts. This remains close to the threshold of ~30 wt% FeO or Fe# >0.40 suggested by molecular dynamics calculations for stable Martian basal melts^32^, but liquids less rich in FeO would therefore be less likely to form a stable BML, especially for a colder and denser residual mantle.

However, this compositional threshold is only indicative, because stability ultimately depends on the melt–solid density contrast, which also varies with the pressure, melt temperature and mantle temperature. If a BML is formed from a less FeO-rich mantle source, its stability would require additional Fe enrichment or enhanced melt production, for example, through very low-degree melting, progressive melt segregation, fractional crystallization, warmer basal temperatures, hydrogen-bearing melting or enrichment in heat-producing elements. Conversely, more FeO-rich sources would promote denser melts and strengthen basal accumulation. For instance, if mantle overturn transported moderately Fe-enriched residual material into the lower mantle, basal melts could have formed from a source that is more FeO rich than the average bulk-Mars mantle, thereby promoting the production and accumulation of dense liquids.

## Formation of a dense basal silicate melt layer

A major implication of our results is that the dense Fe-rich melts generated near the Martian CMB are unlikely to remain trapped as a weakly partially molten assemblage over geological timescales. Their near-neutral to negative buoyancy, combined with low wetting angles, early melt interconnection and low viscosities^32^, should favour efficient melt–solid segregation and downward melt focussing over geological timescales^35^. In a basal layer thicker than 100 km, compaction-driven segregation could concentrate melt towards the base of the mantle, producing a melt-rich and potentially locally fully molten silicate layer, even if some solid residual and compositional stratification may remain^6^.

Our results provide an alternative to the overturn scenario for the origin of the Martian basal low-velocity layer. In classical magma ocean models^36^, a dense basal layer is commonly produced by overturn of an extremely Fe-enriched reservoir generated during fractional crystallization of the primordial magma ocean. Our data suggest that such extreme pre-enrichment is not necessarily required: in fact, low-degree partial melting of a bulk-Mars mantle composition near the CMB can itself generate Fe-rich liquids with densities suitable for downward segregation and accumulation. However, forming an ~150–180-km-thick basal magma layer would require the accumulation of a melt volume equivalent to ~5% of the Martian silicate mantle. Such a volume is unlikely to be produced by localized low-degree melting alone, unless past mantle convection enabled efficient extraction and focussing of melts from a large fraction of the mantle. The required melt volume may instead record large-scale melt segregation during extensive mantle melting or magma ocean crystallization.

Alternatively, if mantle overturn did occur, our results suggest that high-pressure melting was likely limited to low melt fractions, because more extensive melting would generate less Fe-rich and more buoyant liquids (Extended Data Fig. 8). This favours a scenario in which overturn occurred largely in the solid state, followed by remelting during thermal equilibration and mixing of overturned material within the hot lower mantle. Efficient post-overturn mixing may therefore have promoted low-degree melting over a broader lower-mantle region, as suggested by numerical models of mantle mixing after overturn^37,38^. According to our revised solidus, ambient lower-mantle temperatures may have been sufficient to induce partial melting during thermal equilibration and mixing, particularly in the hotter early Martian mantle, without requiring additional heating from below. Moderate Fe enrichment of the overturned material would further favour the production and accumulation of dense melts but is not a prerequisite for melting. Additional heat supplied by a hot or superheated core^39^ could nevertheless have enhanced the extent of melting and melt production, representing an end-member scenario rather than a necessary condition. Beyond Mars, these findings suggest that Fe-rich basal melt layers may be a common and long-lived feature of differentiated terrestrial bodies^40^, with first-order consequences for their thermal, chemical and dynamical evolution.

## Methods

### Sample preparation

A FeO-rich bulk composition representative of the Martian mantle^10,42^ was selected as starting material (Supplementary Table 1). Reagent-grade powders of SiO_2_, Al_2_O_3_, MgO, NaAlSi_3_O_8_ and CaCO_3_ were ground in an agate mortar under ethanol for 2 h to obtain a homogeneous mixture. The FeO-free oxide–carbonate mixture was decarbonated at 1,273 K overnight, after which Fe_2_O_3_ was added. Homogeneous glass spheres (diameter ~2.5–3 mm) were produced at the Institut de Physique du Globe de Paris by melting and quenching the powders in an aerodynamic laser-levitation furnace^44^ under controlled gas mixtures (pure Ar and mixture of CO_2_–H_2_). The resulting glasses have Fe^3+^/Fe_Total_ ratios of ~0.3–0.4 (ref. ^45^), and the compositions determined by electron probe microanalysis are reported in Supplementary Table 1. Water contents were measured by Fourier transform infrared spectroscopy; no OH-bearing bands were detected, and the starting material was considered nominally dry. For high-pressure experiments, the glass spheres were polished into cylindrical pellets and directly inserted into multi-anvil assemblies. Before in situ ultrasonic and synchrotron measurements at beamline BL04B1, samples were equilibrated at high pressure and temperature in a Walker-type multi-anvil press at Institut de Minéralogie, de Physique des Matériaux et de Cosmochimie (IMPMC) to crystallize phase assemblages analogous to the Martian mantle. Tungsten carbide (Hawedia) secondary anvils (32 mm edge length) with 8- and 4-mm truncation edge lengths were used. Pressures up to 17 GPa were generated using 14-mm and 10-mm MgO + Al_2_O_3_ octahedral pressure media surrounded by pyrophyllite. COMPRES-type assemblies^46^ were employed, with a rhenium foil furnace surrounded by a zirconia (ZrO_2_) insulating sleeve. Samples were enclosed in metal capsules to isolate them from the assembly^47^. On the basis of available constraints on Martian mantle oxygen fugacity (see, for example, refs. ^48–51^), we opted for a sealed Re capsule for our synthesis experiments at higher pressure. The samples were equilibrated for 1–2 h under the following pressure–temperature conditions (Supplementary Fig. 10 and Supplementary Table 4): around 17–18 GPa and 1,600 K (ringwoodite stability field), 14–15 GPa and 1,600 K (wadsleyite stability field) and 4 GPa and 1,400 K (olivine stability field). After sintering, each sample was carefully double-side polished to a thickness of 800 µm to 1 mm, achieving a mirror-like surface (~0.5 µm precision) using diamond-coated polishing tools.

### Oxygen fugacity

Mössbauer spectroscopy of the starting glasses^45^ indicates Fe^3+^ abundances corresponding to oxygen fugacities of approximately ΔQFM of +2 to +3 (ref. ^52^), where QFM denotes the quartz–fayalite–magnetite buffer. For in situ ultrasonic and synchrotron experiments, pre-synthesized samples were sealed in rhenium (Re) capsules. In situ melting experiments at SPring-8 provide additional constraints on redox conditions. Oxygen fugacity is influenced by molybdenum foils between the sample and possibly by the presence of boron nitride in the pressure marker. We observe systematic exsolution of metallic Fe^0^, indicating redox conditions more reducing than the iron–wüstite (IW) buffer (Extended Data Figs. 2 and 3). Oxidation of Mo to MoO_2_–MoO_3_ may consume oxygen from the sample, driving reduction of Fe^3+^ to Fe^2+^ and Fe^0^, and probably producing ~1–2 vol.% metallic iron in recovered run products. Additional constraints on the oxygen fugacity can be inferred from phase equilibria in selected run products. In the unmelted sample containing magnesiowüstite, equilibrium considerations indicate oxygen fugacities of approximately IW − 0.5. This estimate is relatively robust because the presence of magnesiowüstite provides a well-defined redox reference. For melt-bearing samples, attempts to estimate the oxygen fugacity using equilibria involving majoritic garnet yield values broadly consistent with those inferred for the magnesiowüstite-bearing assemblage, but with uncertainties comparable to the magnitude of the offset from IW (approximately ±0.5 log units). As a result, the available thermodynamic data do not allow a quantitatively meaningful determination of the oxygen fugacity for the partially molten samples. Nevertheless, the consistent observation of metallic Fe^0^ exsolution across the melting experiments provides a robust qualitative constraint, indicating that redox conditions during melting were at or below the IW buffer. Such reducing conditions are geophysically relevant and consistent with those expected near the Martian CMB, where the oxygen fugacity is buffered close to IW by interaction with an Fe-rich core. At more oxidizing conditions, near or above IW, less iron would be expected to exsolve as metallic Fe^0^ and a larger fraction of the bulk iron budget would remain in the silicate assemblage and melt as Fe^2+^ or Fe^3+^. This could increase slightly the total FeO content of the silicate liquid relative to our reduced experiments, potentially increasing the melt density and further favouring gravitational stability near the CMB. More oxidizing conditions could also modify the Fe–Mg partitioning between olivine and ringwoodite and the melt and will affect the melt fraction and solidus temperature, although the direction and magnitude of these effects are difficult to quantify without externally buffered experiments. Finally, changes in the Fe^2+^/Fe^3+^ ratio may influence the melt structure and therefore properties such as the density and viscosity.

### Ultrasonic measurements at the BL04B1 beamline

All in situ ultrasonic and X-ray diffraction measurements were performed at beamline BL04B1 (SPring-8, Japan) using the SPEED-1500 multi-anvil apparatus, a DIA-type 1,500-ton press. Pressures up to 18 GPa were generated using 14-mm and 10-mm (Mg,Co)O medium octahedral pressure media and with eight cubic tungsten carbide second-stage anvils (Fujilloy, TF05) with 8- and 5-mm truncation edge lengths, respectively. Samples were surrounded by an MgO sleeve, which was surrounded by a cylindrical rhenium (Re) resistance heater with thickness of approximately 25 µm (Extended Data Fig. 1). Two alumina rods were used as a buffer rod for the acoustic wave travel time measurements and as a backing plate to maintain planar geometry during partial melting, respectively. A cold compressed pellet of either NaCl + Au + hexagonal boron nitride (hBN) or MgO + Au + hBN was placed behind the backing plate to improve the hydrostaticity and provide internal pressure calibration using the known equations of state^53–55^. The temperature was monitored using a W_97_Re_3_–W_75_Re_25_ thermocouple, with the hot junction placed adjacent to the pressure marker. After reaching the target pressure, samples were annealed at 1,400 K for 30–60 min to release non-hydrostatic stresses generated during cold compression. X-ray diffraction and ultrasonic measurements were then carried out during stepwise heating to temperatures exceeding the aggregate solidus. The phases and unit cell volumes of the sample and pressure marker were determined by energy-dispersive X-ray diffraction at a fixed diffraction angle of ~6°, calibrated against Au standard reflections at 300 K. The polychromatic X-ray beam was collimated to ~0.10 mm × 0.30 mm, and diffracted photons (30–150 keV) were collected using a multi-channel Ge solid-state detector calibrated against the fluorescence lines of ^55^Fe, ^57^Co and ^133^Ba. Absolute energy calibration was better than ±30 eV per channel. The beam size was rapidly switched in ~0.5 s between the diffraction and imaging modes using a motorized WC flipper slit system^56^, with typical beam sizes of 1.5 mm (vertical) × 3.0 mm (horizontal) for radiographic measurements and 0.2 mm (vertical) × 0.05 mm (horizontal) for X-ray diffraction measurements. The unit cell parameters of major phases were refined using Le Bail profile fitting using Pdindexer^57^ or GSAS with the EXPGUI interface^58,59^.

Sample lengths were determined by X-ray radiography using a high-resolution complementary metal-oxide–semiconductor Hamamatsu C11440 camera coupled to a 30-µm cerium-doped gadolinium aluminium gallium garnet scintillator. The thickness was defined by tracking 2.5-µm Mo foils placed at both ends of the sample, with a spatial resolution of ~1.3 µm per pixel. Compressional (P) and shear (S) wave travel times were measured using the pulse–echo overlap method at resonant frequencies of 30–70 MHz, but also at 40–60 MHz (ref. ^60^). Acoustic pulses were generated using a waveform and pulse generator coupled to a LiNbO_3_ transducer mounted on the rear face of a WC anvil, and a custom three-dimensionally printed interface ensured stable coupling between the transducer and the anvil assembly at high temperature. Waveforms were recorded using a digital oscilloscope, and travel times were determined from reflections at the buffer rod–sample and sample–support interfaces using IgorPro or EchoFinder software^61^. The compressional (*V*_P_) and shear wave (*V*_S_) velocities were then calculated using the relation *V*_P,S_ = 2*L*/*t*_P,S_, where *L* is the length of the sample and *t*_P,S_ is the two-way travel time of P and S waves, respectively. Uncertainties on the P and S wave velocities were estimated by propagating the uncertainties on travel time picking, sample length, pressure and temperature using a Monte Carlo approach. After stress relaxation, measurements were conducted from sub-solidus to super-solidus conditions in temperature steps of 25–50 K, with each step maintained for 5–10 min. At the end of each experiment, the electrical power to the furnace was cut to quench the sample. Among the four experimental runs, two experiments (runs S3631 and S3632) yielded reproducible *V*_P_ and *V*_S_ signals over the full investigated temperature range, whereas runs S3630 and S3565 showed a progressive degradation of the ultrasonic signals above ~1,773–1,873 K, making the velocity data difficult to exploit. For these latter runs, priority was given to in situ X-ray diffraction measurements and the samples were quenched from temperatures above the solidus to preserve high-temperature phase and textural information.

### Temperature gradient and uncertainties

Heat transfer within the multi-layer cell assemblies was modelled using the CellAssembly software package^62^. Modelling was performed for both the in situ 14/8 assembly and the in situ 10/5 assembly (Supplementary Fig. 11), with geometries constrained from post-run *X*–*Z* cross-sections of recovered assemblies. The main sources of uncertainty in the thermal models arise from limited constraints on the thermal conductivity at high pressure and temperature, mechanical relaxation during decompression and assumptions regarding the thermal properties of the sample (peridotitic bulk composition). Temperature fields were calculated for thermocouple readings from 1,773 K to 2,173 K for both assemblies. In all experiments, a systematic thermal gradient is observed within the sample, with a colder region located near the buffer rod and a hotter region towards the centre and the lateral margins of the sample. Reported melt fractions and bulk chemical compositions correspond to the central part of the samples, which is inferred to best represent the maximum experimental temperature conditions. The intra-sample gradient is estimated to be on the order of ~20–50°C. Although local melt pockets and small-scale textural heterogeneities are observed in recovered samples, the large representative areas used for image analysis and chemical measurements suggest that first-order variations in the melt fraction and melt composition remain limited across most samples. Experiment S3630 is an exception, showing two sharply contrasted textural domains; for this run, two liquid populations and two independent melt fraction estimates are therefore reported.

The models indicate that temperature differences between the sample centre and the adjacent thermocouple position are typically ~40–60 K when the sample is correctly centred within the 14/8 assembly. Larger deviations are predicted for the smaller 10/5 assembly, particularly if the sample is offset from the assembly axis, a condition that can be verified by inspection of recovered sample geometries. For the 10/5 configuration, modelled temperature offsets are on the order of ~100–120 K. On the basis of the relative positions of the thermocouple junction and the sample centre, all reported temperatures were corrected by applying offsets of −50°C for experiments using the 14/8 assembly and −110°C for experiments using the 10/5 assembly.

### Further ex situ experiments

Complementary ex situ quench experiments were performed at the Bayerisches Geoinstitut (BGI), using Kawai-type multi-anvil presses equipped with 32-mm WC cubes (4 mm truncation) and 10-mm Cr_2_O_3_-doped MgO octahedra. Two experiments were conducted at pressures of up to 18 GPa in molybdenum capsules (bended foils) with quenching at 2,173 and 2,273 K, reproducing the temperature paths applied during the beamline experiments. In addition to thermocouple measurements, melting temperatures were independently estimated using the Al solubility thermometer in MgO^15^ (Extended Data Fig. 2). At temperatures above the solidus at 18 GPa, quenched textures display clear evidence of interstitial silicate melt, independently confirming the onset of melting at conditions consistent with in situ determinations. The co-existence of silicate melts and metallic Fe^0^ thus provides independent validation of both the low oxygen fugacity and the location of the solidus under deep Martian mantle conditions. Three additional ex situ melting experiments were performed at 19 GPa to investigate the evolution of melt chemistry with increasing degree of melting. These experiments used the starting powder rather than glass spheres; the powder was first equilibrated in a gas-mixing furnace at QFM-2 to QFM-3. Experiments were conducted with graphite capsules, which are expected to transform to diamond at high pressure. A thermocouple was placed in direct contact with the capsule, except for experiment H6050, which was run without a thermocouple because of the high target temperature. Runs were held for 10 min at the target conditions and then quenched. BGI assemblies differed from the in situ configuration and employed an LaCrO_3_ tubular resistance furnace, a zirconia thermal insulator, a D type thermocouple and molybdenum or graphite capsules. Pressure was calibrated using in-run phase transitions as internal markers, including the quartz–coesite–stishovite transition and Fo_80_–Fo_90_ olivine equilibria^63^. Experimental conditions are summarized in Supplementary Table 4.

### Microstructure and chemistry of recovered samples

The textures and mineralogy of all recovered samples were characterized by scanning electron microscopy (SEM) on longitudinal sections oriented parallel to the furnace axis. Samples were embedded in epoxy and polished to a mirror finish. Visual inspection confirmed that cylindrical sample geometries were generally preserved, even at high melt fractions. Sub-solidus samples display micrometre-scale polygonal grains with triple junctions close to 120°, indicative of textural equilibrium (Supplementary Fig. 12). Cracks, where present, are attributed to tensile stresses developed during quenching or decompression. SEM imaging was performed using field-emission instruments (Zeiss Ultra55 at IMPMC, France and LEO 1530 at BGI, Germany) operated at 15–20 kV and working distances optimized for the detection of thin melt films and low-degree melt textures. Phase identification was guided by element mapping of Mg, Al, Si, Ca and Fe. Quenched melts were distinguished from crystalline phases by Fe and Ca enrichment (Supplementary Fig. 4). Modal phase proportions were quantified using two independent approaches: First, backscattered-electron SEM images were processed with ImageJ and MATLAB, and greyscale contrasts were used to estimate phase proportions. Multiple pixel resolutions were tested to ensure robustness, and results are reported as volume percentages, assuming that analysed sections are representative of the bulk. Second, modal abundances were derived by mass balance calculations based on phase compositions measured by electron microprobe analysis. Modal proportions were calculated from the measured compositions of the co-existing phases and the bulk composition. Uncertainties on modal abundances (and corresponding melt fractions) were estimated as ±1 standard deviation from the mass balance calculations and are shown as horizontal error bars in Fig. 3 and Extended Data Figs. 5 and 7. Analyses were conducted using a Cameca SXFiveTactis (IMPMC) and a JEOL JXA-8200 (BGI) at 15 kV accelerating voltage and 10–20 nA beam current. Major elements (Si, Fe, Al, Mg and Ca) were quantified using natural and synthetic standards. Mineral phases were analysed with a focussed beam (~1 µm), whereas bulk compositions were verified using defocussed beam conditions (20–30 µm) (Supplementary Table 5).

### High-pressure density modelling of Fe-rich melts

Liquid densities were independently calculated using three different high-pressure equations of state, following the formulations of Asimow et al.^23^, Wakabayashi and Funamori^24^ and Jing and Karato^25^. This multi-model approach was used to evaluate the sensitivity of the calculated densities to the choice of liquid EoS. In the Asimow formulation, the bulk melt composition was decomposed into mineralogical liquid end-members with independently constrained elastic and thermal properties. Compression along the reference isentrope was calculated using third- or fourth-order Birch–Murnaghan equations, depending on the parameters available for each liquid end-member:

$$\begin{array}{lll}{P}_{{\rm{S}}}(V)=3{K}_{0{\rm{S}}}\,f{\left(1+2f\,\right)}^{5/2}\left\{1+\frac{3}{2}\left({K}_{{\rm{S}}}^{{\prime} }-4\right)\right.\\\qquad\qquad\left.f+\frac{3}{2}\left[{K}_{0{\rm{S}}}{K}_{{\rm{S}}}^{{\prime}{\prime} }+\left({K}_{{\rm{S}}}^{{\prime} }-4\right)\left({K}_{{\rm{S}}}^{{\prime} }-3\right)+\frac{35}{9}\right]{f}^{\,2}\right\}\end{array}$$

where *f* is the Eulerian strain parameter $f=\frac{1}{2}\left[{\left(\frac{{V}_{0}}{V}\right)}^{2/3}-1\right]$ and ${K}_{0{\rm{S}}},\,K_{{\rm{S}}}^{{\prime}}\,\mathrm{and}\,K^{{\prime}{\prime} }_{{\rm{S}}}$ are the ambient-condition isentropic bulk modulus and its first and second pressure derivative. When fourth-order parameters were unavailable, the ${f}^{\,2}$ contribution was omitted. The total pressure was obtained by adding a Mie–Grüneisen thermal–pressure contribution:

$$P\left(V,T\right)={P}_{{\rm{S}}}\left(V\right)+\gamma \left(V\right)\rho {C}_{V}\left[T-{T}_{{\rm{S}}}\left(V\right)\right]$$

where $\gamma \left(V\right)={\gamma }_{0}{\left(V/{V}_{0}\right)}^{q}$. Here, ${\gamma }_{0}$ is the Grüneisen parameter at the reference volume ${V}_{0}$, *q* is the dimensionless exponent describing the volume dependence of γ, ρ is the density at volume *V*, ${C}_{V}$ is the isochoric heat capacity and ${T}_{{\rm{S}}}\left(V\right)$ is the temperature along the reference isentrope. Reference temperatures were defined separately for each liquid end-member. Two alternative compositional decompositions were evaluated. Model 1 used diopside, anorthite, fayalite, forsterite and enstatite components, whereas model 2 used diopside, anorthite, fayalite, hedenbergite and enstatite, including the negative hedenbergite correction required by the compositional solution. Model 1 yielded systematically much higher densities than the other EoS formulations and was therefore retained as a sensitivity test but not used for the preferred density comparison (Supplementary Table 6).

The Wakabayashi–Funamori formulation describes progressive compression and structural densification of silicate liquids using compositionally averaged partial molar volumes, thermal expansivities and bulk moduli. The pressure was calculated from the compression parameter together with a quasi-harmonic thermal–pressure term of the form $\alpha {K}_{T}\left(T-{T}_{0}\right)$. The Jing–Karato formulation treats the liquid as a mixture of deformable hard-sphere components. The zero-pressure molar volume was calculated from the partial molar volumes of the oxide components, whereas its pressure dependence was determined by the packing, deformation and volume dependence of the effective sphere diameters. These procedures were applied either along isotherms (Extended Data Fig. 8) or along a selected Martian mantle temperature profile slightly above the solidus (Fig. 6 and Supplementary Fig. 9). This areotherm was based on thermal conditions proposed near the Martian CMB^6,7^ and was consistent with the temperature at which ~5% melting was observed in our experiments. For each model, dry-melt density was obtained from the ratio of the bulk molar mass to the calculated pressure- and temperature-dependent molar volume:

$${\rho }_{{\rm{melt}}}=\frac{\sum _{i}{C}_{i}{M}_{i}^{{\rm{EM}}}}{\sum _{i}{C}_{i}{V}_{i}^{{\rm{EM}}}}$$

where ${C}_{i},{M}_{i}^{{\rm{EM}}}$, and ${V}_{i}^{\,{\rm{EM}}}$ are the molar proportion, molar mass and pressure- and temperature-dependent molar volume of component *i*, respectively. Density uncertainties were estimated from 2,000 Monte Carlo realizations sampling the available published 1*σ* EoS parameter uncertainties, while composition, pressure and temperature were held fixed (Supplementary Table 6).

### Residual solid aggregate density model

The density and elastic properties of residual solid aggregates were calculated using a thermoelastic modelling procedure inspired by Zhou et al.^64^ and described in detail in Supplementary Methods. Measured ringwoodite and garnet–majorite compositions from the 18–19-GPa experiments were first projected onto ideal end-member components. Ringwoodite was treated as a binary Mg_2_SiO_4_–Fe_2_SiO_4_ solid solution, whereas garnet–majorite was projected onto pyrope, almandine, grossular, Mg-majorite and Fe-majorite end-members by constrained least-squares fitting. End-member densities and elastic moduli were thermally corrected, mixed according to the calculated solid-solution proportions and compressed using finite-strain EoS. Ringwoodite and garnet–majorite were then combined into a residual aggregate using mass balance-constrained phase proportions and Voigt–Reuss–Hill averaging. Uncertainties in thermoelastic parameters were propagated by Monte Carlo sampling. Sensitivity tests were also performed by allowing small non-ideal excess molar volumes in the solid solutions.

## Online content

Any methods, additional references, Nature Portfolio reporting summaries, source data, extended data, supplementary information, acknowledgements, peer review information; details of author contributions and competing interests; and statements of data and code availability are available at https://doi.org/10.1038/s41561-026-02104-z.

## Supplementary information

Supplementary Information Supplementary Figs. 1–13 and Discussion.

Supplementary Tables Supplementary Tables 1–6.

## Source data

Source Data Figs. 2–6 and Extended Data Figs. 2–8 Source data for Figs. 2–6 and Extended Data Figs. 2–8.

## Data Availability

All data supporting the conclusions of this study are included in the article and its Supplementary Information. Raw data and Excel tables are available via Zenodo at https://doi.org/10.5281/zenodo.21791659 (ref. ^65^). Source data are provided with this paper.

## Code availability

The custom Python scripts used to process the data, perform the calculations and generate the figures are available via Zenodo at https://doi.org/10.5281/zenodo.21791659 (ref. ^65^).
