# Supplementary material for: Experimental constraints on a long-lived magma layer at the Martian core–mantle boundary
Source: Nat Geosci. 2026 Sep 18;19(11):1276–83. doi: 10.1038/s41561-026-02104-z (PMC13739749; doi:10.1038/s41561-026-02104-z)
Supplement: Supplementary file 1 [file 41561_2026_2104_MOESM1_ESM.pdf]

nature geoscience

Supplementary information https://doi.org/10.1038/s41561-026-02104-z

Experimental constraints on a long-lived magma layer at the Martian core–mantle boundary

In the format provided by the authors and unedited

Supplementary information:

Melting phase diagram

Our experiments establish the solidus of a Martian mantle composition up to 18 GPa (Fig. 4), delineating the stability of the major mantle phases across the pressure range of Mars' interior. *In situ* X-ray diffraction was used to track phase transitions during progressive melting, allowing *in situ* monitoring of melting progress (Supplementary Table 3). Experiments were quenched above the solidus, at different stages of partial melting and the resulting textures and residual mineral assemblages were characterized by scanning electron microscopy (SEM) (Extended Data Fig. 3-4). Together, these observations define a melting phase diagram for a FeO-enriched pyrolitic composition representative of the Martian mantle. At pressures around 8 GPa, the phase assemblage resembles terrestrial peridotite and is dominated by olivine, clinopyroxene, orthopyroxene and garnet [1–5]. Melting initiates in orthopyroxene, followed by clinopyroxene and garnet, while olivine remains the liquidus-defining phase. Above ~8–9 GPa, orthopyroxene disappears, consistent with previous high-pressure experimental studies [3,6–8]. Between ~9 and 16 GPa, melting is controlled by clinopyroxene and majoritic garnet, whereas olivine and its high-pressure polymorphs remain comparatively refractory. Above ~16 GPa, clinopyroxene is no longer stable at the solidus, yielding a garnet–olivine (or wadsleyite/ringwoodite) cotectic [7]. Garnet thus exerts strong control over melt compositions across a wide pressure interval. The wadsleyite–ringwoodite transition near ~16 GPa marks a major change in Mars' deep mantle, with these polymorphs constituting nearly half of the crystalline assemblage. At the highest pressures explored (17–18 GPa), only ringwoodite and majoritic garnet (and possibly magnesiowüstite [2]) remain as residual crystalline phases above the solidus. This phase topology indicates that deep Martian melting is governed by pyroxene breakdown at intermediate depths and garnet–ringwoodite persistence toward the core–mantle boundary (CMB).

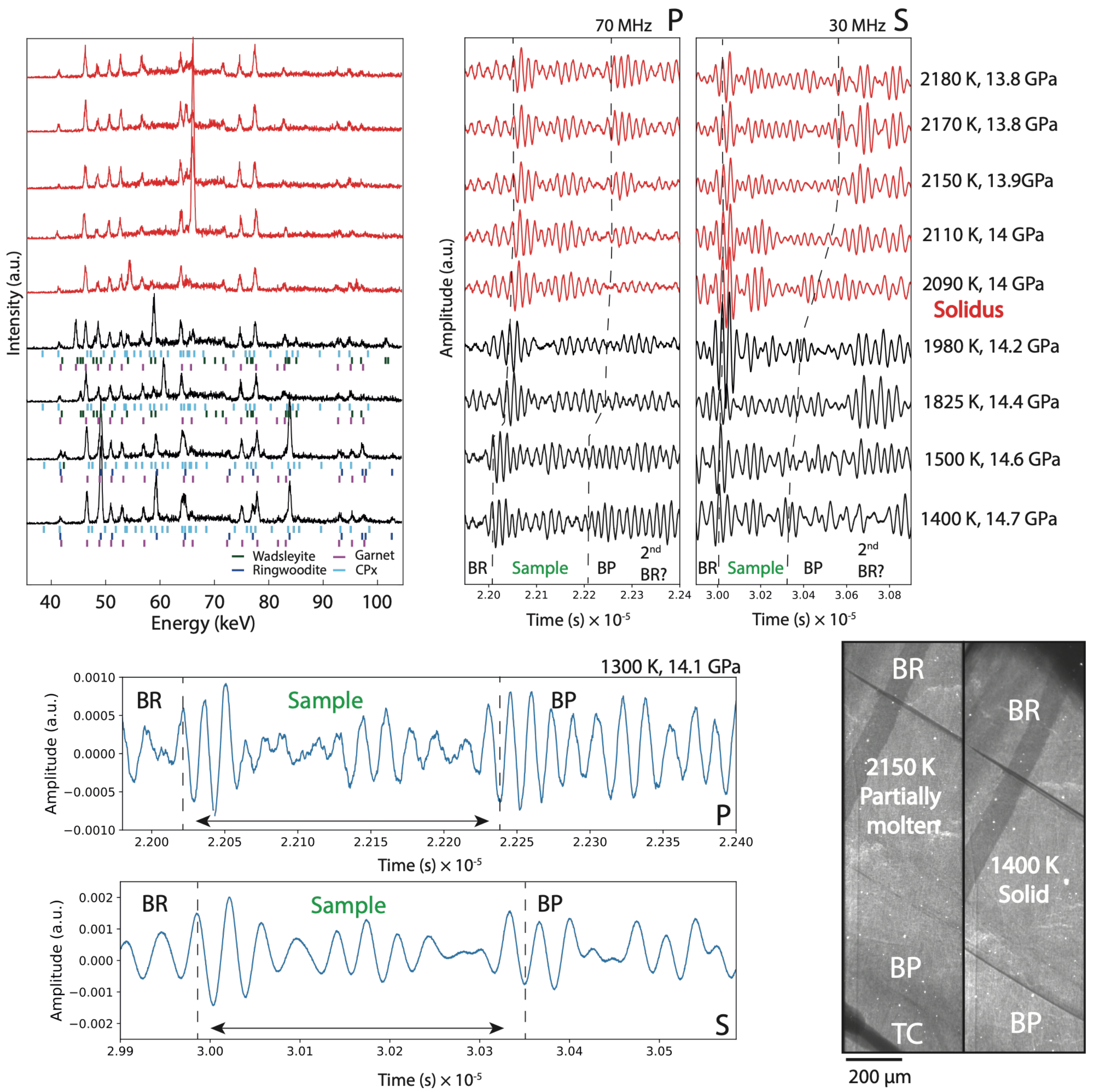
Intensity (a.u.)
Energy (keV)
Wadsleyite
Ringwoodite
Garnet
CPx
70 MHz P
30 MHz S
Amplitude (a.u.)
BR
Sample
BP
2nd BR?
Time (s) × 10-5
2180 K, 13.8 GPa
2170 K, 13.8 GPa
2150 K, 13.9GPa
2110 K, 14 GPa
2090 K, 14 GPa
Solidus
1980 K, 14.2 GPa
1825 K, 14.4 GPa
1500 K, 14.6 GPa
1400 K, 14.7 GPa
1300 K, 14.1 GPa
P
S
2150 K
Partially
molten
1400 K
Solid
TC
200 µm

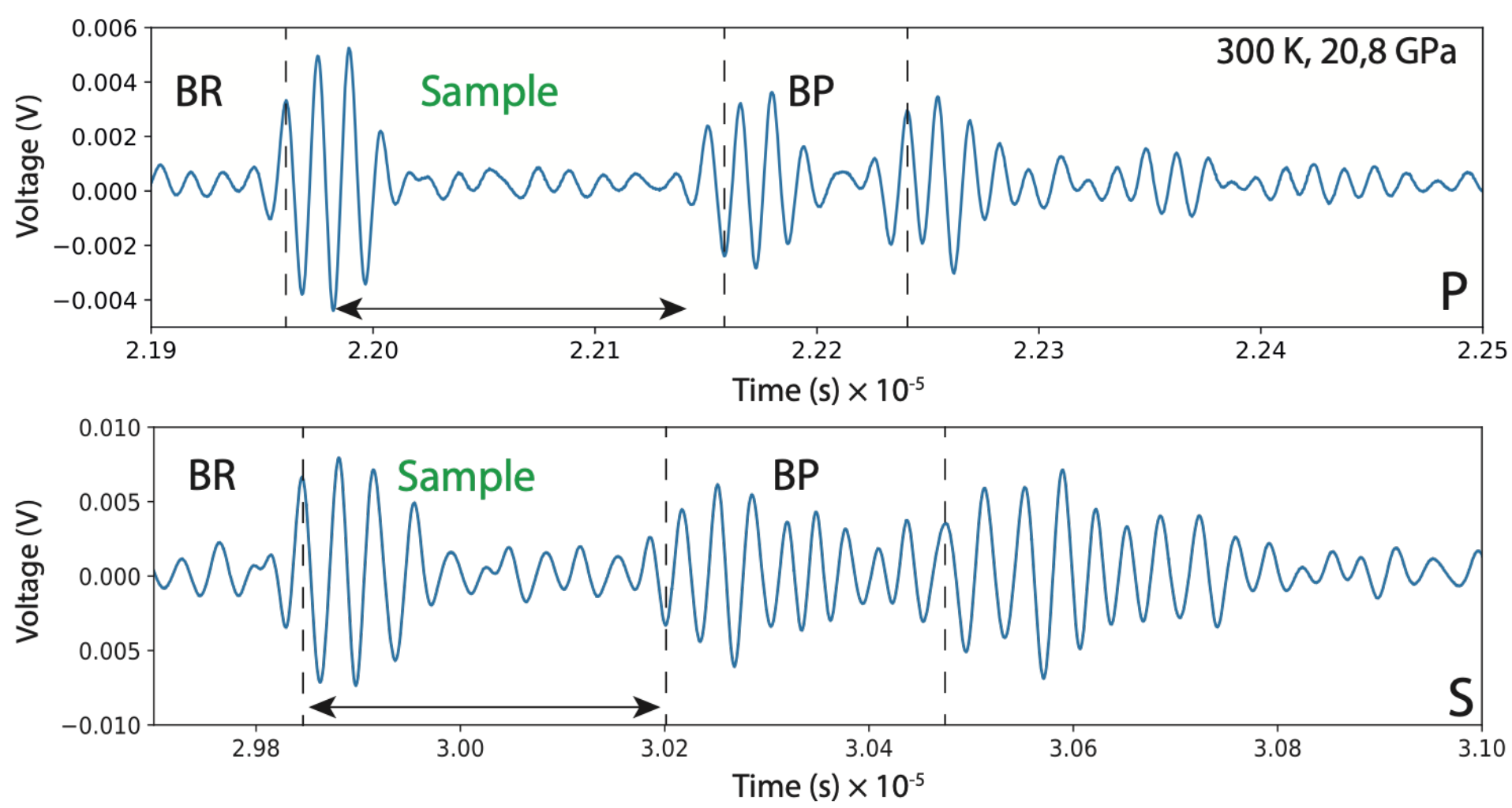
300 K, 20,8 GPa
Voltage (V)
BR
Sample
BP
P
S
Time (s) × 10-5

Fig S1. High-pressure ultrasonic measurements during melting experiments on a Martian mantle assemblage at ~14 GPa. Upper-left panel: *in situ* energy-dispersive X-ray diffraction patterns collected during stepwise heating. Tick marks indicate the main reflections of wadsleyite, ringwoodite, garnet and clinopyroxene. Upper-middle and upper-right panels: time-domain ultrasonic waveforms for compressional V_P at 70 MHz and shear V_S at 30 MHz, acquired from subsolidus to supersolidus conditions between 1400 and 2180 K at 14.7–13.8 GPa. The approximate position of the solidus is indicated by the transition from black to red waveforms. Middle panels: representative V_P and V_S waveforms collected at 1300 K and 14.1 GPa, showing the buffer-rod (BR), sample and backing-plate (BP) echoes used to determine travel times. Middle-right panel: X-ray radiographs of the sample assembly under solid conditions at 1400 K and after partial melting at 2150 K, showing the buffer rod, sample, backing plate and thermocouple position. Interfaces between BR, sample and BP are marked by 2-μm-thick molybdenum foils. Bottom panels: reference V_P and V_S waveforms collected at room temperature and 20.8 GPa, illustrating the echo geometry before high-temperature measurements.

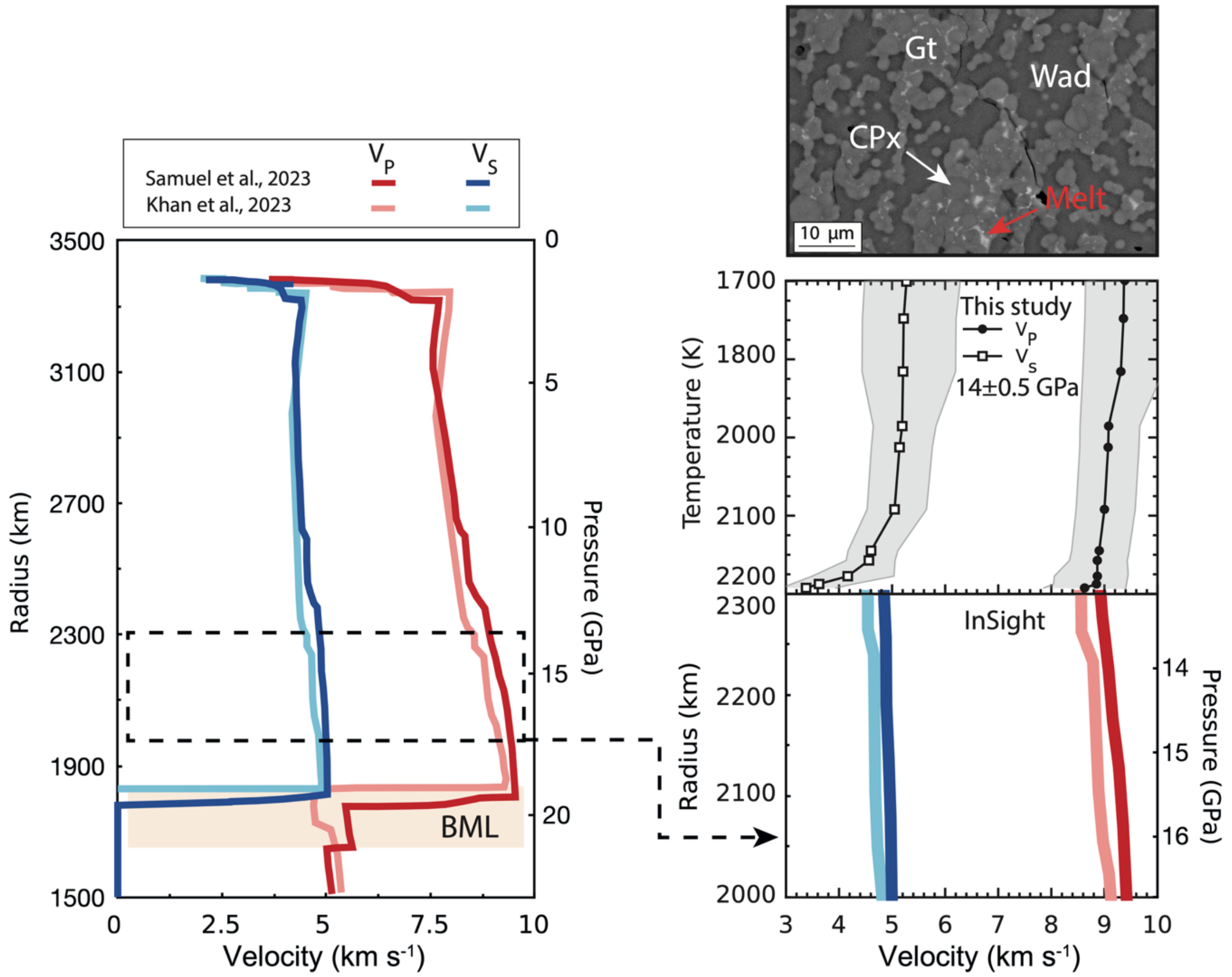

Fig S2. Comparison between experimental ultrasonic velocities and InSight-derived seismic profiles for the Martian lower mantle. Left panel: V_P and V_S profiles from the InSight-based interior models of Samuel et al. [9] and Khan et al. [10], with the shaded region indicating the proposed Martian seismic low-velocity basal magma layer (BML) and the dashed box showing the pressure–depth interval corresponding to the 14 ± 0.5 GPa experiments. Right panels: experimental V_P and V_S measured in this study at 14 ± 0.5 GPa as a function of temperature, compared with the corresponding depth interval of the InSight-derived models. Error bars represent propagated ±1 s.d. uncertainties on ultrasonic velocities. The BSE image illustrates the recovered partially molten assemblage, with interstitial melt between garnet (Gt), wadsleyite (Wad), and clinopyroxene (CPx). The experimental data show that incipient melting produces a marked reduction in V_S, whereas V_P is less affected and remains close to the modelled lower-mantle values. This comparison should be interpreted qualitatively because ultrasonic attenuation and velocity reductions are frequency dependent.

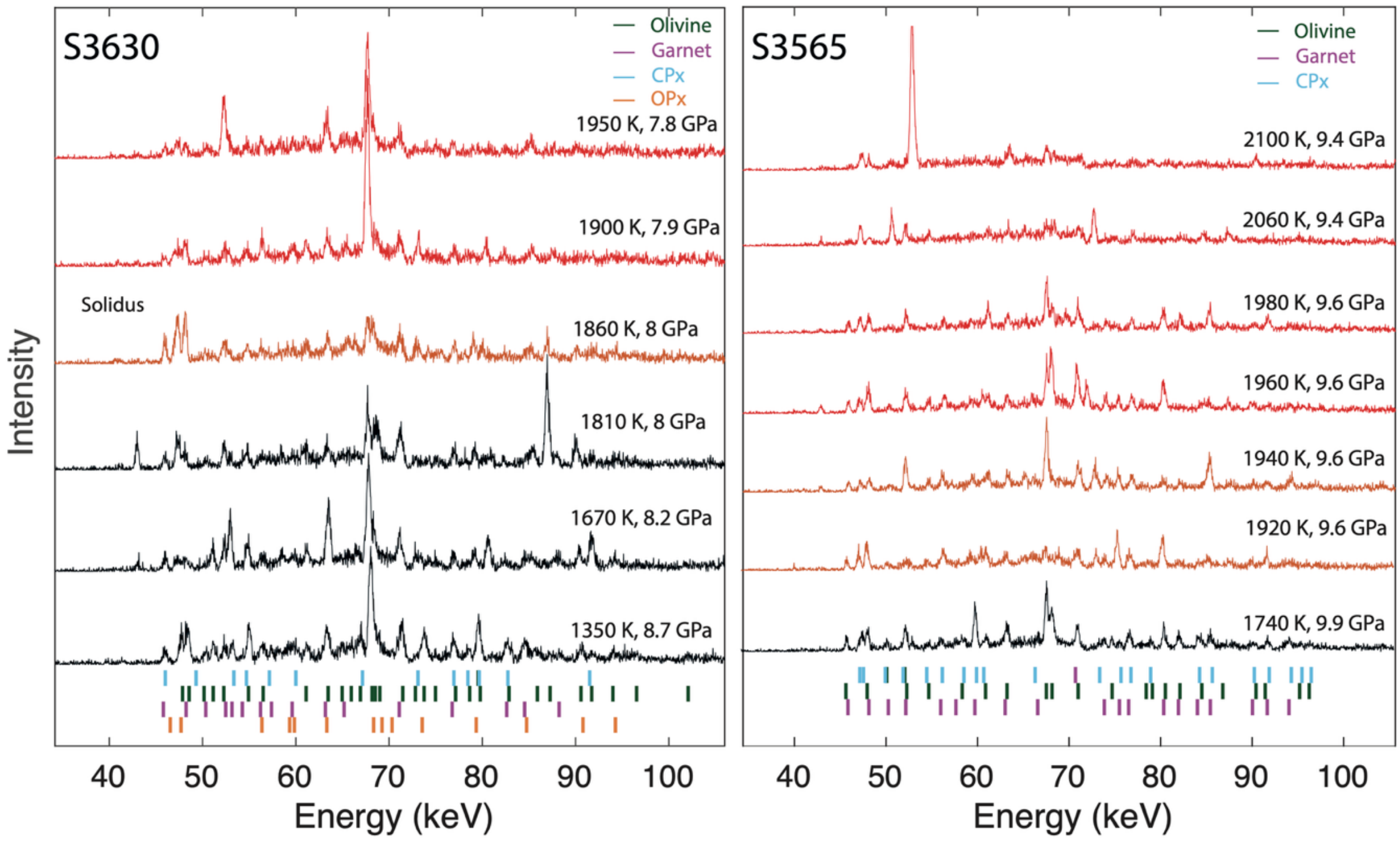

Fig S3. *In situ* energy-dispersive X-ray diffraction (ED-XRD) patterns for runs S3630 and S3565 at high pressure. Representative diffraction sequences are shown for runs S3630 and S3565. Black spectra correspond to fully crystalline subsolidus samples, with sharp Bragg reflections from olivine, garnet, clinopyroxene and orthopyroxene. Orange spectra refer to temperatures near the possible onset of melting, although the diffraction evidence remains ambiguous. Red spectra correspond to higher-temperature conditions where melting is inferred from rapid temporal variations in Bragg peak intensities, progressive peak broadening, disappearance or weakening of selected Bragg reflections, and increased diffuse background. These changes reflect loss of crystalline order, grain motion and development of melt during heating. Together with the ultrasonic observations, these ED-XRD sequences provide in situ constraints on the onset of melting in Fe-rich Martian mantle analogues.

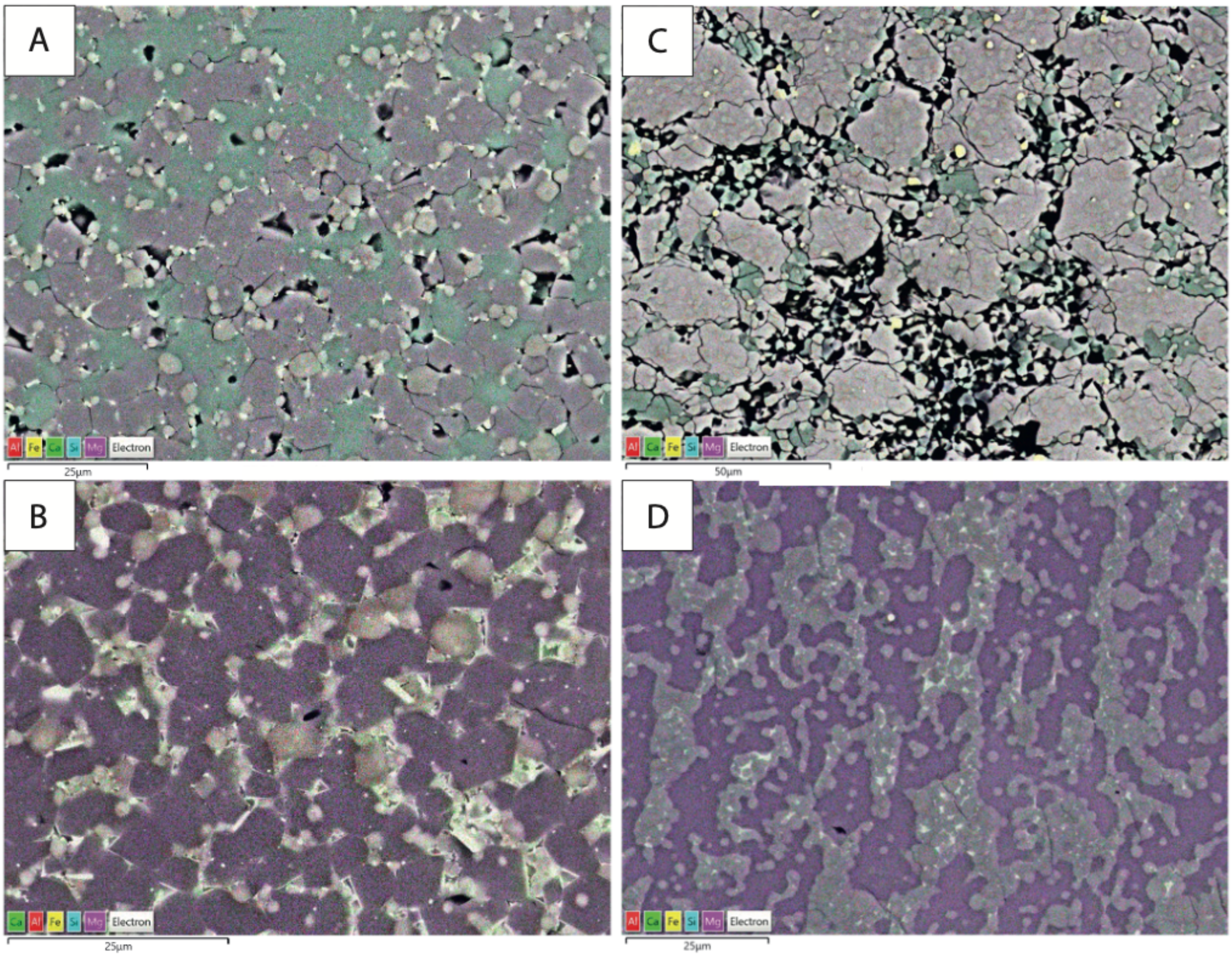

Fig S4. Scanning electron microscopy chemical maps. (A,B) Two regions of sample S3630 containing low and moderate melt fractions, respectively. At 8–10 GPa, clinopyroxene (Cpx; green) is abundant near the solidus, whereas olivine is shown in violet and garnet in orange–red. The melt is enriched in Ca and Fe, consistent with partial melting of a clinopyroxene-bearing assemblage. (C) Sub-solidus assemblage MA-14 at 14 GPa, showing decreasing clinopyroxene abundance and increasing garnet abundance. (D) Partially molten sample S3632, containing <15% melt and dominated by residual phases. The elements and colours used in each map are indicated in the lower-left corner. "Electron" denotes the superposition of the elemental maps on the BSE image, which highlights grain boundaries.

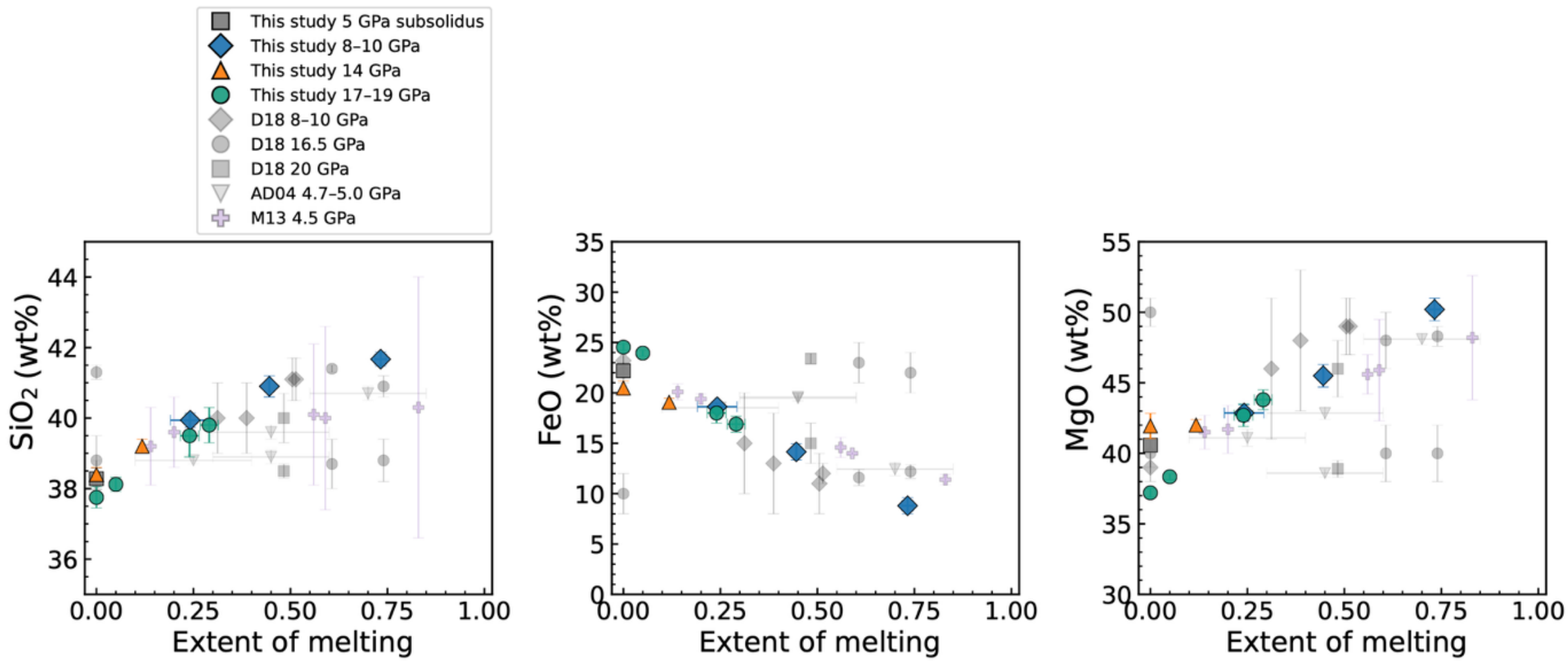

Fig S5. Olivine and polymorph compositions as a function of melt fraction. Major-element compositions of olivine, wadsleyite and ringwoodite from this study are compared with published Martian mantle melting experiments [1,2,4]. Oxide contents are shown as wt% versus extent of melting. At high pressure, wadsleyite and ringwoodite host a major fraction of the Mg–Fe budget of the residual assemblage. Their FeO–MgO evolution with increasing melt fraction is consistent with early extraction of Fe-rich low-degree liquids and progressive enrichment of the residual olivine polymorphs in Mg. Vertical error bars represent ±1 s.d. analytical uncertainties calculated from multiple electron microprobe spot analyses of each phase (n = number of spot analyses; values are reported in Supplementary Table 5). Horizontal error bars represent ±1 s.d. uncertainties on melt fractions estimated from mass-balance calculations.

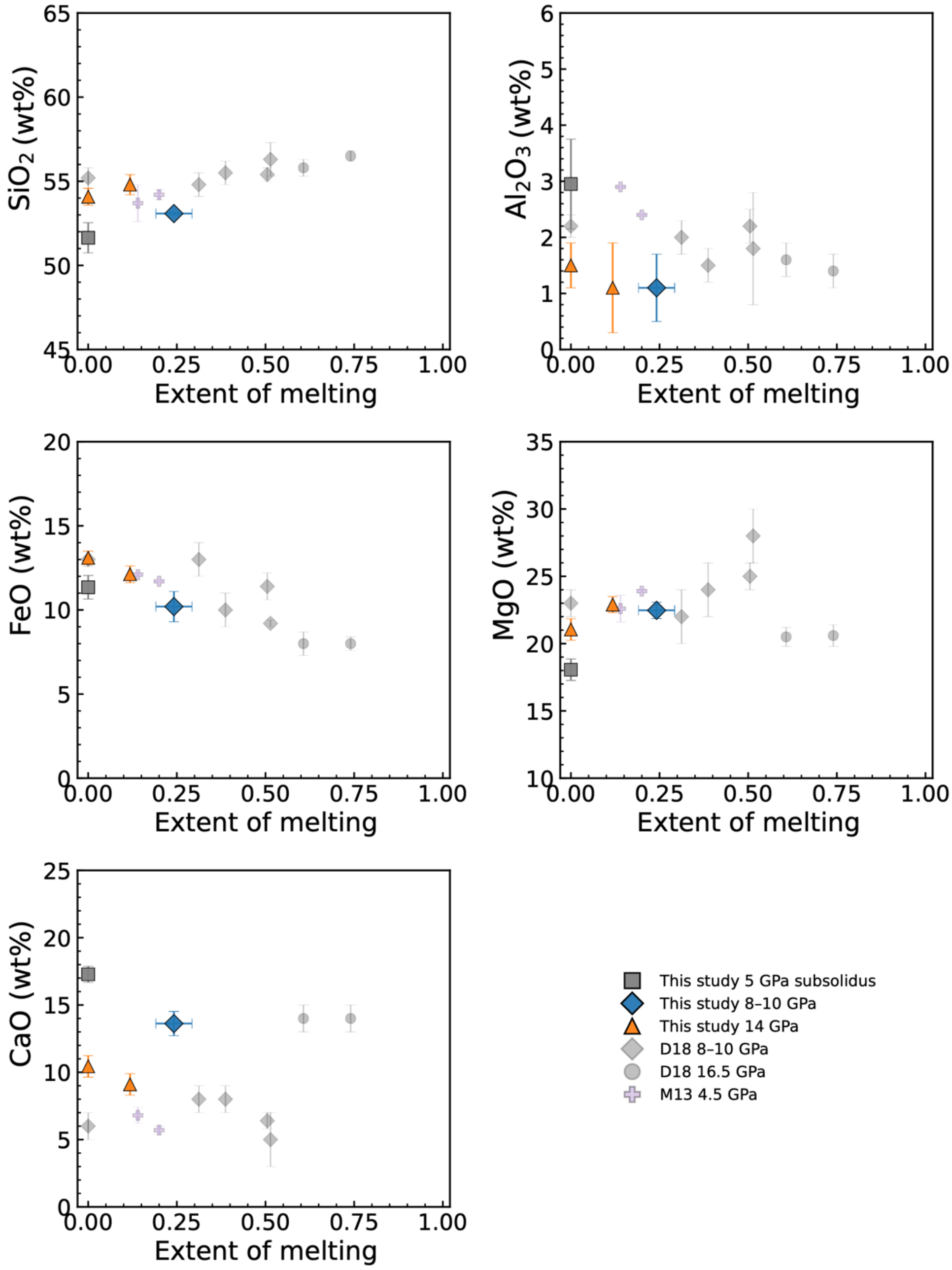

Fig S6. Clinopyroxene compositions as a function of melt fraction. Major-element compositions of clinopyroxene from this study are compared with published Martian mantle melting experiments [1,2,4]. Oxide contents are shown as wt% versus extent of melting. Clinopyroxene is present at lower pressures but becomes progressively less important with increasing pressure and is absent from the highest-pressure melting assemblages, consistent with the transition toward garnet-majorite and olivine polymorph dominated residues at deep Martian mantle conditions. Vertical error bars represent ±1 s.d. analytical uncertainties calculated from multiple electron microprobe spot analyses of each phase (n = number of spot analyses; values are reported in Supplementary Table 5). Horizontal error bars represent ±1 s.d. uncertainties on melt fractions estimated from mass-balance calculations.

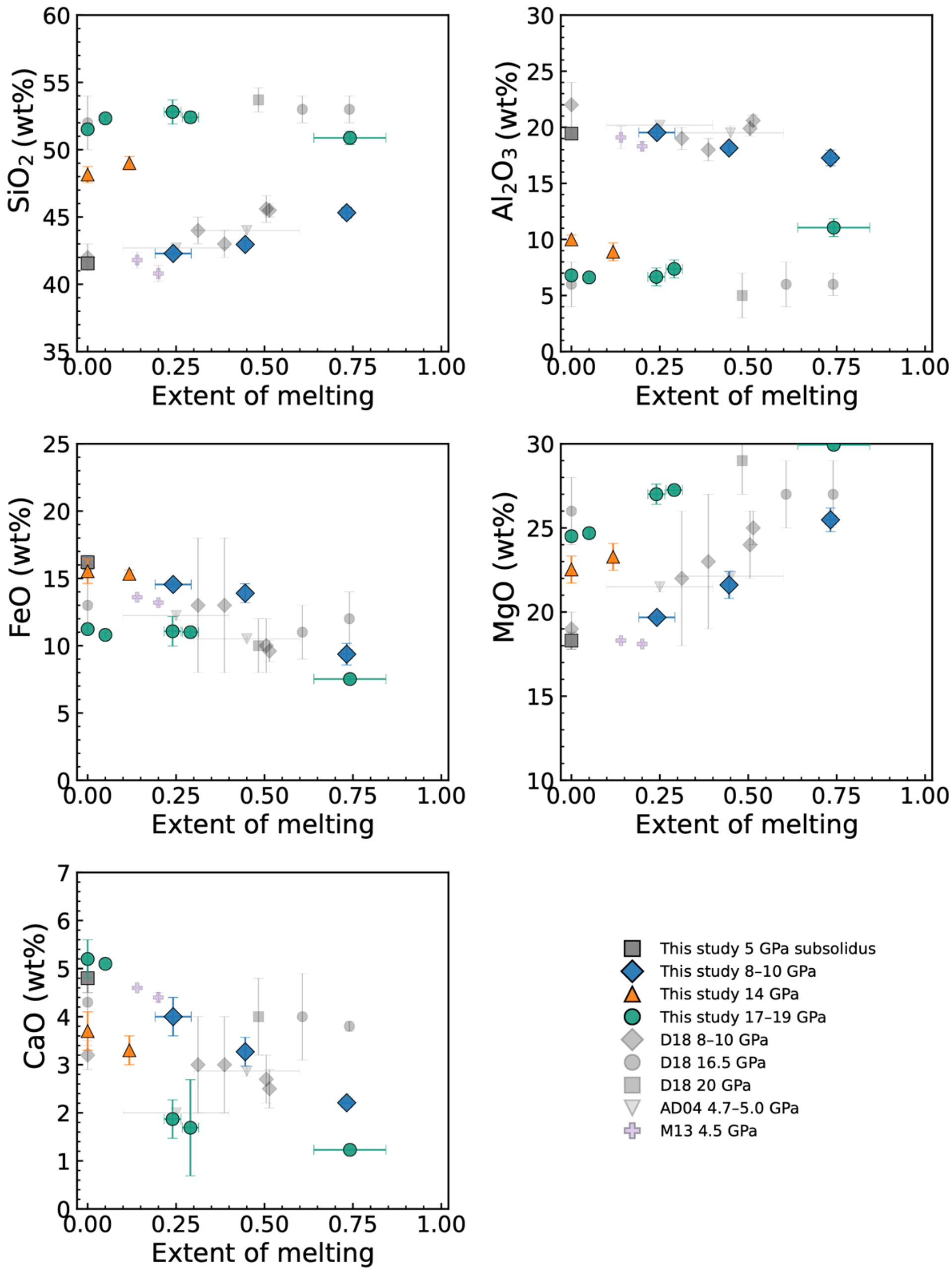

Fig S7. Garnet compositions as a function of melt fraction. Major-element compositions of garnet and majoritic garnet from this study are compared with previous Martian mantle melting experiments [1,2,4]. Oxide contents are shown as wt% versus extent of melting. With increasing pressure, garnet becomes more majoritic and remains an important host for Ca and Al in the residual assemblage. The evolution of FeO and MgO with melt fraction indicates progressive Fe–Mg redistribution during melting, contributing to the production of Fe-rich low-degree liquids. Vertical error bars represent ±1 s.d. analytical uncertainties calculated from multiple electron microprobe spot analyses of each phase (n = number of spot analyses; values are reported in Supplementary Table 5). Horizontal error bars represent ±1 s.d. uncertainties on melt fractions estimated from mass-balance calculations.

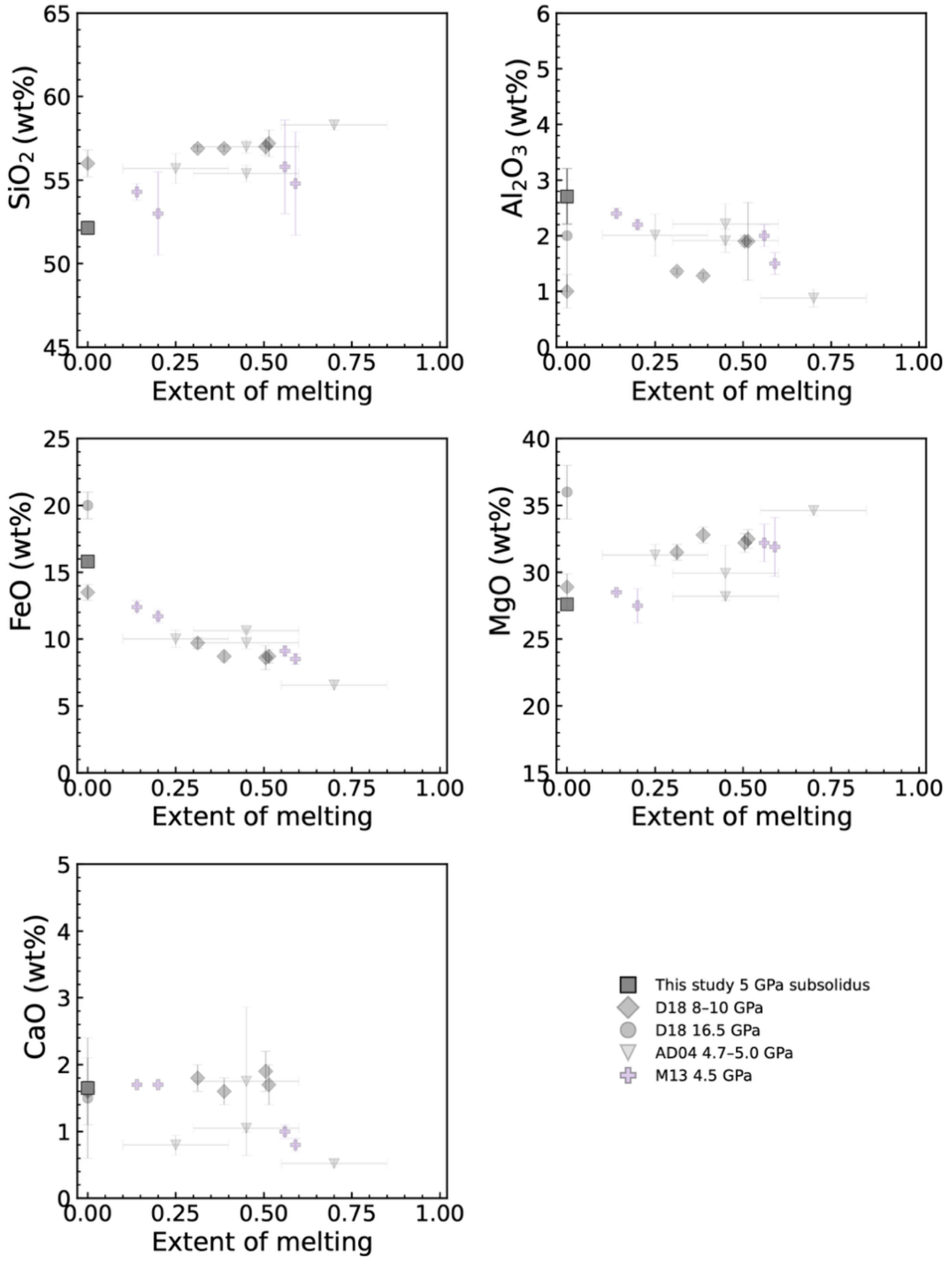

Fig S8. Orthopyroxene compositions as a function of melt fraction. Major-element compositions of orthopyroxene from this study are compared with previous Martian mantle melting experiments [1,2,4]. Oxide contents are shown as wt% versus extent of melting. Orthopyroxene is restricted to lower-pressure assemblages and is not observed in the deepest melting experiments of this study. Its disappearance with increasing pressure, together with the loss of clinopyroxene, marks the transition from pyroxene-bearing residues to high-pressure assemblages dominated by garnet-majorite and olivine polymorphs. Vertical error bars represent ±1 s.d. analytical uncertainties calculated from multiple electron microprobe spot analyses of each phase (n = number of spot analyses; values are reported in Supplementary Table 5). Horizontal error bars represent ±1 s.d. uncertainties on melt fractions estimated from mass-balance calculations.

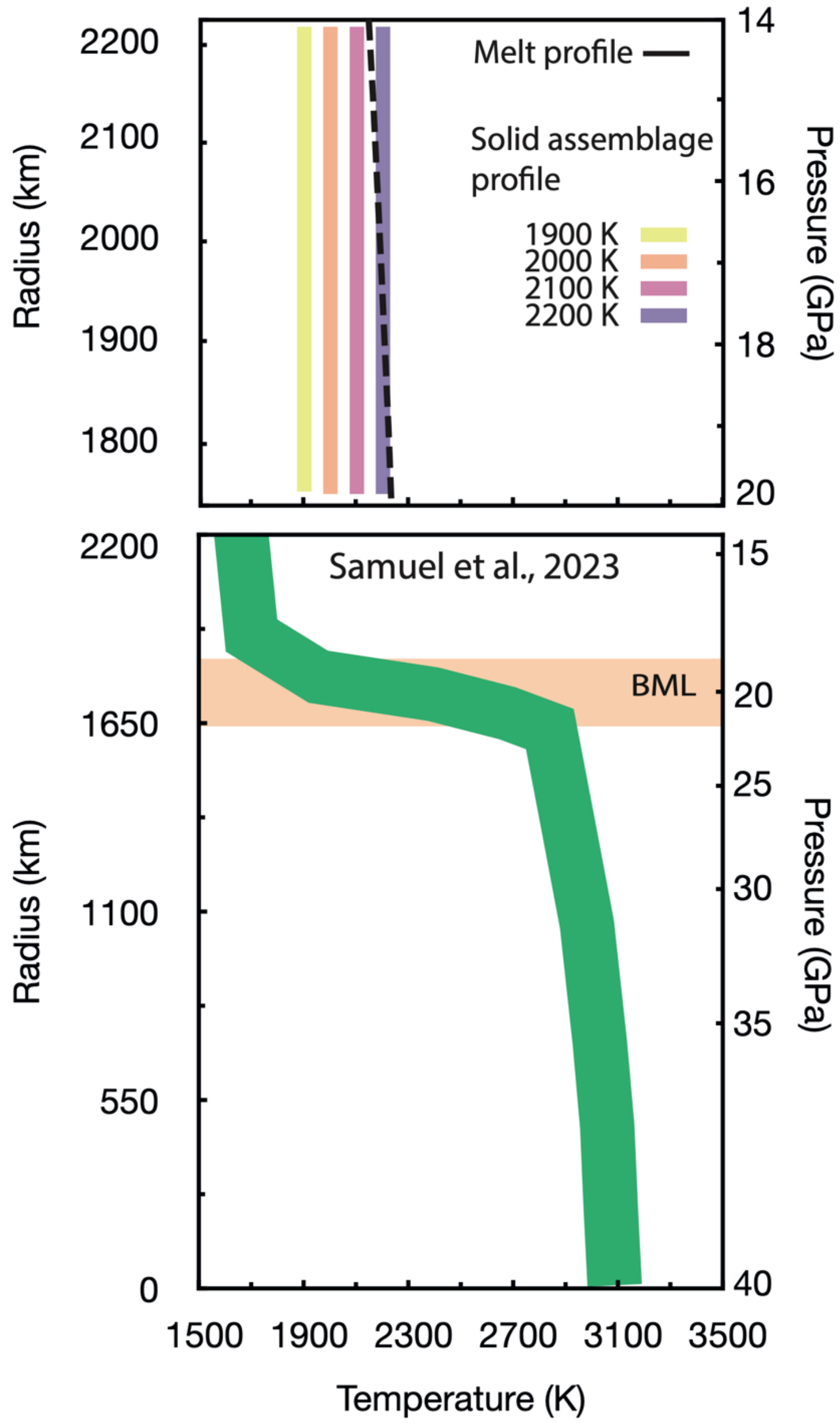

Fig S9. Temperature profile used to calculate the density of the Fe-rich melt with Fe# ≈ 0.5 over the 16–21 GPa pressure range. Top panel: Temperature profile adopted in this study for density calculations, shown as a function of pressure. The black line represents the selected melt-temperature profile, which is consistent with the experimental production temperature of the ~5% melt in sample S3631 at approximately 18 GPa. The coloured lines indicate alternative temperature profiles used for the coexisting solid assemblage, corresponding to basal-mantle temperatures of 1900, 2000, 2100, and 2200 K. Bottom panel: Temperature profiles from recent Mars interior models constrained by InSight seismic and geodetic data, plotted as a function of planetary radius. The green band corresponds to the range of modelled Martian mantle temperatures reported by Samuel et al. [9]. These models predict temperatures of approximately 1900–2300 K near the base of the mantle, within the expected basal magma layer region, supporting the temperature range adopted in this study.

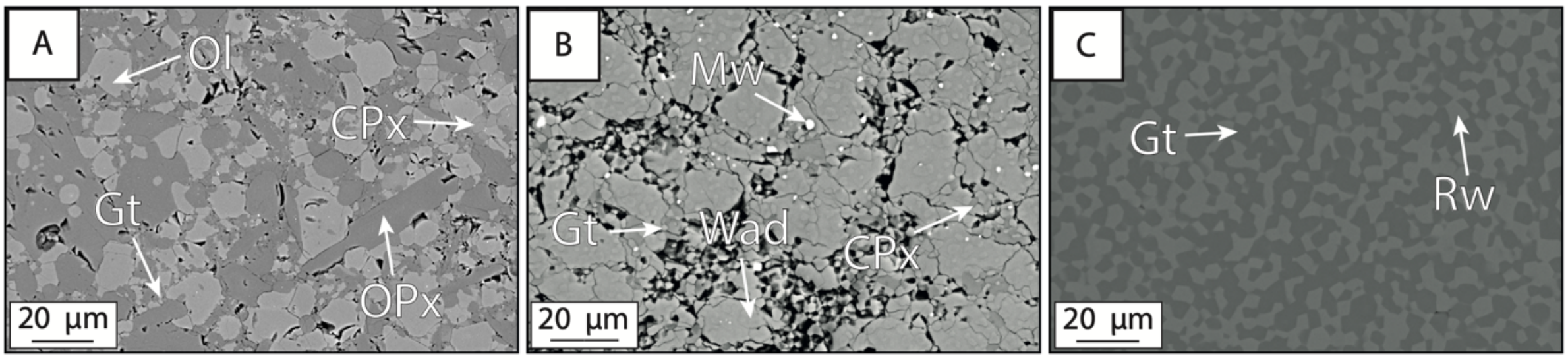

Fig S10: **Backscattered electron images of sub-solidus mineralogical assemblages synthesized for ultrasonic experiments.** A) MA-5: The shallow-mantle experiment at 5 GPa consists of olivine (Ol), pyroxene (CPx + OPx) and garnet (Gt). B) MA-14: The mid-mantle phase assemblage synthesized at 14 GPa contains wadsleyite (Wad), clinopyroxene (CPx), garnet (Gt), magnesiowüstite (Mw) and metallic iron (Fe^0). C) MA-17: The deep mantle phase assemblage synthesized at 17 GPa contains ringwoodite (Rw) and garnet (Gt). The black areas are voids in the samples formed during sample polishing prior to SEM analyses and were not present during the high pressure and high temperature experiments.

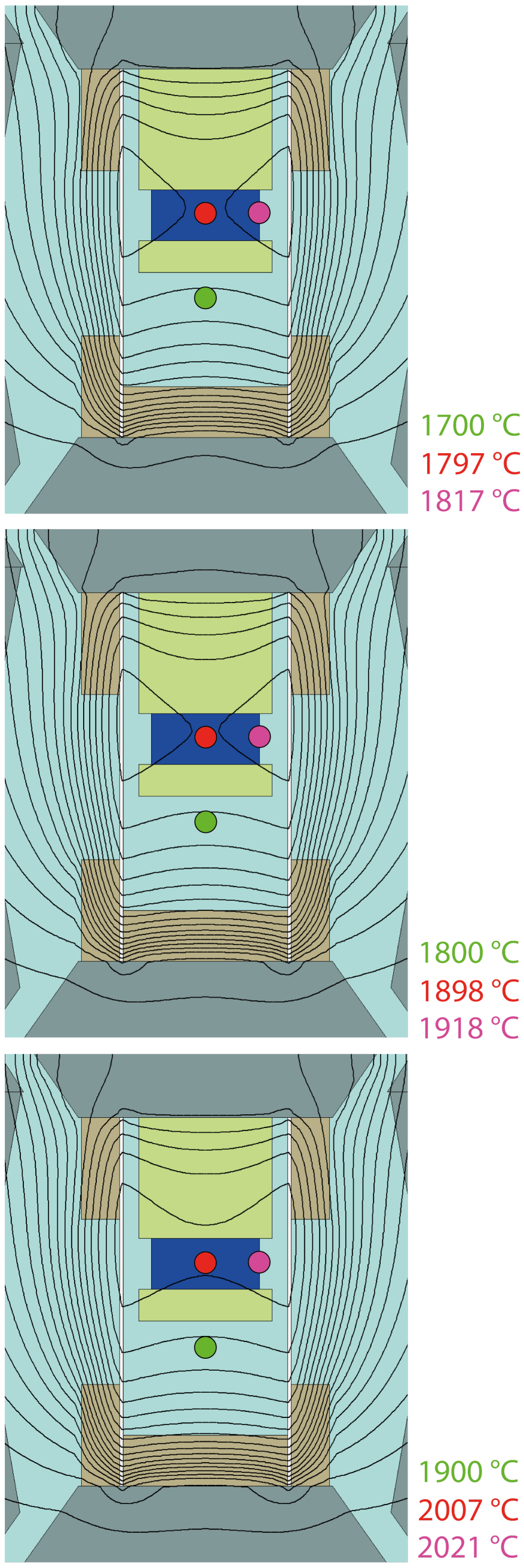

Fig S11. Schematic thermal structure of the 10/5 multi-anvil high-pressure assembly used for ultrasonic measurements. Cross-sections show modelled temperature fields at nominal furnace setpoints of 1700 °C, 1800 °C and 1900 °C. Temperature distributions were calculated using the Cell Assembly software [11], highlighting the development of strong axial thermal gradients within the sample region. Isotherms are spaced by 100 °C. The experimental stack comprises, from the centre outward: Al_2O_3 buffer rods, a pre-synthesised Martian mantle assemblage, pressure markers (MgO, Au and BN), a soft MgO medium, a (Mg,Co)O pressure medium, and a rhenium heater. The green circle marks the position of the thermocouple hot junction, whereas the red circles indicate calculated temperatures within the sample.

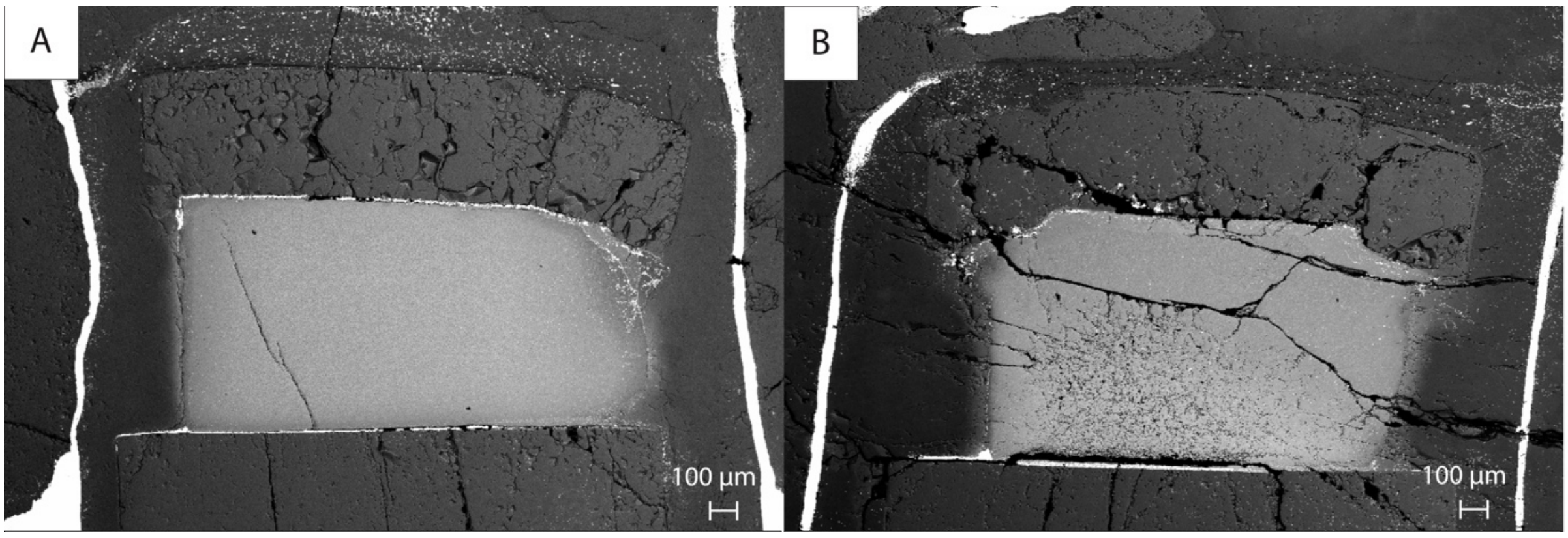

Fig S12. Backscattered electron (BSE) images of recovered samples from ultrasonic experiments. (A) Sample S3631 recovered from approximately 18 GPa, showing the central capsule region after partial melting. (B) Sample S3632 recovered from approximately 14 GPa, showing the corresponding partially molten assemblage.

Volumetric constraints from the basal magma layer

We estimated the fraction of the Martian mantle that must melt in order to generate a basal magma layer (BML) of the thickness inferred from InSight-informed geophysical models. We follow the geometric configuration proposed by [9,10], in which the basal melt layer is located immediately above the core–mantle boundary (CMB) and extends radially from ≈1650 km to 1830 km from the centre of Mars, corresponding to a thickness of ~180 km.

Mars is approximated as a series of concentric spherical shells. We adopt a mean Martian radius of 3,390 km, a metallic core radius of approximately 1,650 km and an average crustal thickness of approximately 50 km. The outer radius of the silicate mantle is therefore taken as 3,340 km.
The total volume of the silicate mantle, V_{mantle}, is calculated as the volume of a spherical shell bounded by radii of 1,650 and 3,340 km:

$$V_{mantle} = \frac{4}{3}\pi(3340^3 - 1830^3)$$

The volume of the basal magma layer, V_{BML}, is computed using the same spherical shell formulation, bounded by radii of 1650 km and 1830 km:

$$V_{BML} = \frac{4}{3}\pi(1830^3 - 1650^3)$$

Using these values, the total silicate mantle volume is 1.373×10^{11} km^3, while the basal magma layer volume is 6.854×10^9 km^3. The inferred basal magma ocean therefore represents approximately 5 % of the Martian silicate mantle volume. This value represents the melt volume required to form the BML. The corresponding volume of mantle source that must undergo partial melting would be larger and would depend on the average melt fraction and the efficiency of melt extraction and focusing.

<u>Density model for the residual solid aggregate</u>

Objective of the model

The objective of the model is to calculate the density and elastic properties of a residual solid aggregate composed of ringwoodite and garnet-majorite as a function of pressure and temperature. The aggregate density is then compared with liquid density estimates over the pressure interval 16–21 GPa.

The model proceeds in four steps: (1) projection of measured phase compositions onto ideal endmembers, (2) calculation of thermoelastic properties for each solid solution, (3) compression of each phase using finite-strain equations of state, and (4) combination of ringwoodite and garnet-majorite into an aggregate using mass balance and Voigt-Reuss-Hill averaging.

The nominal model assumes ideal solid-solution mixing of molar volumes. A sensitivity test is performed by allowing a constant excess molar volume of ±0.5% and ±1.0% for the solid solutions.

Input compositions

The model uses three compositions: the bulk starting composition, the measured ringwoodite composition, and the measured garnet-majorite composition.

Oxide compositions used in the model for S3631

Oxide	Starting composition (wt%)	Ringwoodite (wt%)	Garnet-majorite (wt%)
SiO_2	44.44	37.75	51.52
Al_2O_3	3.10	0.00	6.80
FeO	18.34	24.54	11.23
MgO	31.24	37.20	24.51
CaO	2.48	0.00	5.20
Na_2O	0.40	0.00	0.00

All oxide compositions are first converted to cation proportions and normalized to 12 oxygens. For an oxide i, the number of moles is calculated as:

$$n_i = \frac{w_i}{M_i}$$

where w_i is the oxide weight percent and M_i is the molar mass of the oxide.

The total number of oxygens is:

$$N_O = 2n_{\mathrm{SiO_2}} + 3n_{\mathrm{Al_2O_3}} + n_{\mathrm{FeO}} + n_{\mathrm{MgO}} + n_{\mathrm{CaO}} + n_{\mathrm{Na_2O}}$$

Each cation abundance is then normalized to 12 oxygens:

$$C_j^{12O} = C_j \frac{12}{N_O}$$

where C_j is the unnormalized number of moles of cation j.

Endmember projection

Ringwoodite projection

Ringwoodite is projected onto two ideal endmembers:

$$\mathrm{Fo}_{ring} = \mathrm{Mg_2SiO_4}$$

$$\mathrm{Fa}_{ring} = \mathrm{Fe_2SiO_4}$$

The Fe-Mg ratio is calculated from the 12-oxygen-normalized cation abundances:

$$X_{\mathrm{Fa}} = \frac{\mathrm{Fe}}{\mathrm{Fe} + \mathrm{Mg}}$$

$$X_{\mathrm{Fo}} = 1 - X_{\mathrm{Fa}}$$

Thus, the ringwoodite solid solution is written as:

$$\mathrm{Ringwoodite} = X_{\mathrm{Fo}}\mathrm{Fo}_{ring} + X_{\mathrm{Fa}}\mathrm{Fa}_{ring}$$

Garnet-majorite projection

Garnet-majorite is projected onto five ideal endmembers:

$$\mathrm{Py} = \mathrm{Mg_3Al_2Si_3O_{12}}$$

$$\mathrm{Alm} = \mathrm{Fe_3Al_2Si_3O_{12}}$$

$$\mathrm{Gr} = \mathrm{Ca_3Al_2Si_3O_{12}}$$

$$\mathrm{MgMaj} = \mathrm{Mg_4Si_4O_{12}}$$

$$\mathrm{FeMaj} = \mathrm{Fe_4Si_4O_{12}}$$

The garnet-majorite composition is projected using constrained least squares. The measured cation vector is:

$$\mathbf{b} = \begin{bmatrix} \mathrm{Si} \\ \mathrm{Al} \\ \mathrm{Mg} \\ \mathrm{Fe} \\ \mathrm{Ca} \end{bmatrix}$$

and the endmember stoichiometry matrix is:

$$\mathbf{A} = \begin{bmatrix} 3 & 3 & 3 & 4 & 4 \\ 2 & 2 & 2 & 0 & 0 \\ 3 & 0 & 0 & 4 & 0 \\ 0 & 3 & 0 & 0 & 4 \\ 0 & 0 & 3 & 0 & 0 \end{bmatrix}$$

with columns corresponding to [Py,Alm,Gr,MgMaj,FeMaj].

The endmember fraction vector $\mathbf{x}$ is obtained by minimizing:

$$\min_{\mathbf{x}} \|\mathbf{Ax} - \mathbf{b}\|^2$$

subject to:

$$x_i \geq 0$$

$$\sum_i x_i = 1$$

The projection residual is:

$$\mathbf{r} = \mathbf{Ax} - \mathbf{b}$$

This least-squares projection provides a flexible description because all measured cations are fitted simultaneously. This projection is a simplified compositional representation of the analysed garnet-majorite. It does not imply that the crystal is physically composed of discrete pure endmembers, but provides an internally consistent way to assign thermoelastic properties from ideal endmember data.

Garnet-majorite endmember stoichiometry used for the least-squares projection.

Endmember	Formula	Si	Al	Mg	Fe	Ca
Py	$Mg_3Al_2Si_3O_{12}$	3	2	3	0	0
Alm	$Fe_3Al_2Si_3O_{12}$	3	2	0	3	0
Gr	$Ca_3Al_2Si_3O_{12}$	3	2	0	0	3
MgMaj	$Mg_4Si_4O_{12}$	4	0	4	0	0
FeMaj	$Fe_4Si_4O_{12}$	4	0	0	4	0

Molar masses of endmembers

The molar mass of each ideal endmember is calculated from atomic masses:

$$M_i = \sum_j n_{ij}\, m_j$$

where n_{ij} is the number of atoms of element j in endmember i, and m_j is the atomic mass.

Ideal endmembers used in the model.

Endmember	Formula	Molar mass expression
Fo_ring	Mg_2SiO_4	$2M_{\mathrm{Mg}} + M_{\mathrm{Si}} + 4M_{\mathrm{O}}$
Fa_ring	Fe_2SiO_4	$2M_{\mathrm{Fe}} + M_{\mathrm{Si}} + 4M_{\mathrm{O}}$
Py	$Mg_3Al_2Si_3O_{12}$	$3M_{\mathrm{Mg}} + 2M_{\mathrm{Al}} + 3M_{\mathrm{Si}} + 12M_{\mathrm{O}}$
Alm	$Fe_3Al_2Si_3O_{12}$	$3M_{\mathrm{Fe}} + 2M_{\mathrm{Al}} + 3M_{\mathrm{Si}} + 12M_{\mathrm{O}}$
Gr	$Ca_3Al_2Si_3O_{12}$	$3M_{\mathrm{Ca}} + 2M_{\mathrm{Al}} + 3M_{\mathrm{Si}} + 12M_{\mathrm{O}}$
MgMaj	$Mg_4Si_4O_{12}$	$4M_{\mathrm{Mg}} + 4M_{\mathrm{Si}} + 12M_{\mathrm{O}}$
FeMaj	$Fe_4Si_4O_{12}$	$4M_{\mathrm{Fe}} + 4M_{\mathrm{Si}} + 12M_{\mathrm{O}}$

Calculated endmember fractions used in the model.

**Phase**	**Endmember**	**Fraction**
Ringwoodite	Fo_ring	0.7299
Ringwoodite	Fa_ring	0.2701
Garnet-majorite	Py	0.0989
Garnet-majorite	Alm	0.0558
Garnet-majorite	Gr	0.1339
Garnet-majorite	Mg_Maj	0.5840
Garnet-majorite	Fe_Maj	0.1273

Garnet-majorite least-squares fit quality.

Cation	Measured cations per 12 O	Fitted cations per 12 O	Residual
Si	3.71174	3.71133	-0.00041
Al	0.57739	0.57733	-0.00005
Mg	2.63241	2.63271	+0.00030
Fe	0.67663	0.67693	+0.00030
Ca	0.40140	0.40170	+0.00030

Two-phase mass-balance result and oxide residuals.

Quantity	Weight fraction	Fitted wt%	Residual wt%
ringwoodite	0.5271		
garnet-majorite	0.4729		
SiO_2		44.2617	-0.1783
Al_2O_3		3.2157	+0.1157
FeO		18.2458	-0.0942
MgO		31.1990	-0.0410
CaO		2.4590	-0.0210

Thermoelastic parameters

Each endmember is assigned a reference density, bulk modulus, shear modulus, pressure derivatives, temperature derivatives, and thermal expansion parameters.

Thermoelastic parameters used for each endmember

Symbol	Meaning	Unit
ρ_0	Reference density at 300 K and 0 GPa	g cm^{-3}
K_S	Adiabatic bulk modulus	GPa
G	Shear modulus	GPa
$\partial K_S / \partial P$	Pressure derivative of bulk modulus	dimensionless
$\partial^2 K_S / \partial P^2$	Second pressure derivative of bulk modulus	GPa^{-1}

Symbol	Meaning	Unit
$\partial K_S / \partial T$	Temperature derivative of bulk modulus	GPa K^{-1}
$\partial G / \partial P$	Pressure derivative of shear modulus	dimensionless
$\partial^2 G / \partial P^2$	Second pressure derivative of shear modulus	GPa^{-1}
$\partial G / \partial T$	Temperature derivative of shear modulus	GPa K^{-1}
a_0, a_1, a_2	Thermal expansion parameters	see equation below

The thermal expansion parameterization is:

$$\alpha(T) = a_0 \times 10^{-4} + a_1 \times 10^{-8} T + \frac{a_2}{T^2}$$

The integrated thermal expansion between $T_{\text{ref}} = 300\,\text{K}$ and T is:

$$I(T) = a_0 \times 10^{-4}(T - T_{\text{ref}}) + \frac{1}{2} a_1 \times 10^{-8}\left(T^2 - T_{\text{ref}}^2\right) + a_2\left(\frac{1}{T_{\text{ref}}} - \frac{1}{T}\right)$$

Thermal correction of endmembers

Each endmember is thermally corrected before solid-solution mixing. The thermally corrected density is:

$$\rho_{0,i}(T) = \rho_{0,i}\exp[-I_i(T)]$$

The bulk and shear moduli are corrected linearly with temperature:

$$K_{S,i}(T) = K_{S,i}(300) + \left(\frac{\partial K_S}{\partial T}\right)_i (T - 300)$$

$$G_i(T) = G_i(300) + \left(\frac{\partial G}{\partial T}\right)_i (T - 300)$$

The pressure derivatives are corrected as:

$$K'_{S,i}(T) = K'_{S,i}(300)\exp[I_i(T)]$$

$$G'_i(T) = G'_i(300)\exp[I_i(T)]$$

The second pressure derivatives are kept unchanged:

$$K''_{S,i}(T) = K''_{S,i}(300)$$

$$G''_{i}(T) = G''_{i}(300)$$

Solid-solution mixing

For each endmember, the thermally corrected molar volume is:

$$V_i(T) = \frac{M_i}{\rho_i(T)}$$

The ideal mixed molar volume is:

$$V_{\text{mix}}^{\text{ideal}}(T) = \sum_i x_i\, V_i(T)$$

where x_i is the endmember fraction. The nominal model assumes $V_{\text{excess}} = 0$, so:

$$V_{\text{mix}}(T) = V_{\text{mix}}^{\text{ideal}}(T)$$

The thermally corrected solid-solution density is then:

$$\rho_{\text{mix}}(T) = \frac{\sum_i x_i\, M_i}{V_{\text{mix}}(T)}$$

The elastic moduli of the solid solution are mixed linearly over endmember fractions:

$$K_{S,\text{mix}}(T) = \sum_i x_i\, K_{S,i}(T)$$

$$G_{\text{mix}}(T) = \sum_i x_i\, G_i(T)$$

The pressure derivatives and second derivatives are also mixed linearly:

$$K'_{S,\text{mix}}(T) = \sum_i x_i\, K'_{S,i}(T)$$

$$G'_{\text{mix}}(T) = \sum_i x_i\, G'_{i}(T)$$

$$K''_{S,\text{mix}}(T) = \sum_i x_i\, K''_{S,i}(T)$$

$$G''_{\text{mix}}(T) = \sum_i x_i\, G''_{i}(T)$$

This mixing approach is an approximation. It treats the solid solutions as ideal with respect to molar volume in the nominal model and neglects possible non-ideal mixing. In the current implementation, the elastic moduli of the solid solutions are mixed linearly over endmember molar fractions. Voigt-Reuss-Hill averaging is not applied at the solid-solution endmember level; it is applied only later when ringwoodite and garnet-majorite are combined into the final aggregate.

<u>Excess molar volume sensitivity test</u>

To evaluate the effect on the volume of the non-ideal solid-solution behaviour, a constant excess molar volume term can be introduced:

$$V_{\mathrm{mix}}(T) = V_{\mathrm{mix}}^{\mathrm{ideal}}(T)(1 + f_{\mathrm{excess}})$$

where f_{excess} is the fractional excess volume.

The sensitivity test uses:

$$f_{\mathrm{excess}} = -0.010, -0.005, 0, +0.005, +0.010$$

corresponding to:

$$V_{\mathrm{excess}} = -1.0\%,\ -0.5\%,\ 0\%,\ +0.5\%,\ +1.0\%.$$

The same excess-volume fraction is applied to both the ringwoodite and garnet-majorite solid solutions. It is therefore a test on the solid-solution molar volumes before calculating the aggregate density, not a direct excess-volume correction applied to the final aggregate. In the reported sensitivity test, the constant excess-volume option is used. The pairwise excess-volume is implemented in the code but all pairwise interaction terms are set to zero and are not used in the nominal model.

A positive excess volume increases the solid-solution molar volume and decreases density:

$$f_{\mathrm{excess}} > 0 \Rightarrow \rho_{\mathrm{mix}} \downarrow$$

A negative excess volume decreases molar volume and increases density:

$$f_{\mathrm{excess}} < 0 \Rightarrow \rho_{\mathrm{mix}} \uparrow$$

The excess-volume sensitivity test shows that imposing non-ideal solid-solution effects of ± 0.5 to ± 1.0 on the residual ringwoodite–garnet-majorite aggregate produces only moderate shifts in calculated solid density (Fig. S13). Across this range, the solid aggregate remains close to the Fe# ≈ 0.5 liquid density at 18–20 GPa, while Fe-richer liquids remain denser than the residual mantle. Thus, the inferred near-neutral to negative buoyancy of dense Fe-rich melts is not strongly affected by reasonable uncertainties in solid-solution excess molar volume.

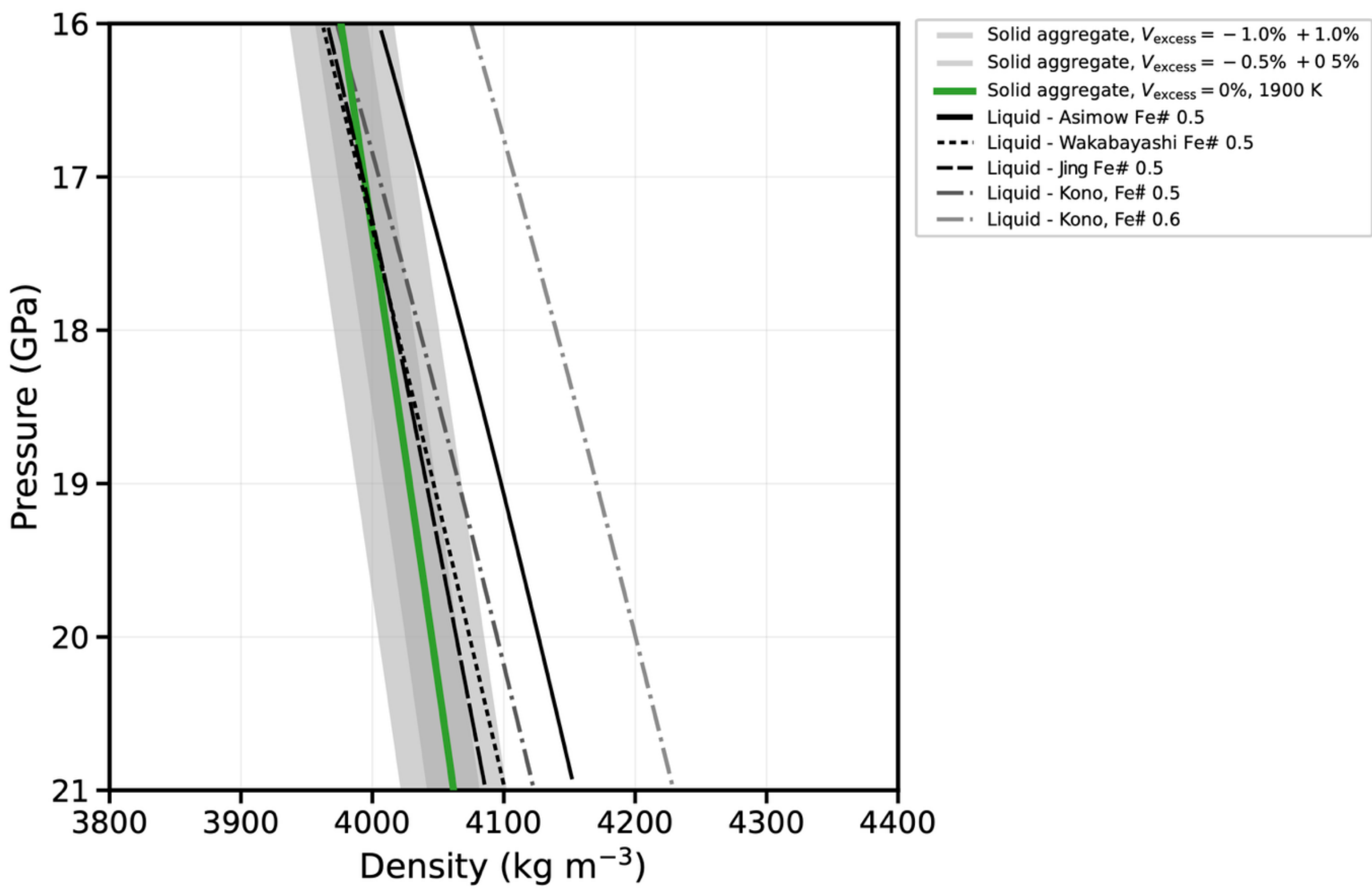

Fig S13: Sensitivity of residual solid density to solid-solution excess molar volume and comparison with Fe-rich melt densities. Density–pressure profiles are shown for the residual ringwoodite–garnet-majorite aggregate at 1900 K, calculated for ideal solid solutions ($V_{excess} = 0$; green curve) and with imposed excess molar volume variations of ± 0.5 and ± 1.0 (grey envelopes). Liquid density estimates for Fe-rich melts are shown for comparison using the Asimow [12], Wakabayashi/Funamori [13], Jing/Karato [14] and Kono [15] formulations, including Kono models [15] with Fe# = Fe/(Fe + Mg) = 0.5 and 0.6. Grey envelopes represent the 16th–84th percentiles obtained from 10,000 Monte Carlo realizations propagating uncertainties in the thermoelastic parameters.

Finite-strain equation of state

The model uses an Eulerian finite-strain formulation. The finite strain variable is:

$$\varepsilon = \frac{1}{2}\left[1 - \left(\frac{\rho}{\rho_0(T)}\right)^{2/3}\right]$$

Equivalently, after solving for ε, the density is:

$$\rho(P,T) = \rho_0(T)(1-2\varepsilon)^{3/2}$$

The pressure is calculated from finite-strain coefficients. In the third-order case:

$$P(\varepsilon) = -(1-2\varepsilon)^{5/2}\left[C_1\varepsilon + \frac{1}{2}C_2\varepsilon^2\right]$$

In the fourth-order case:

$$P(\varepsilon) = -(1-2\varepsilon)^{5/2}\left[C_1\varepsilon + \frac{1}{2}C_2\varepsilon^2 + \frac{1}{6}C_3\varepsilon^3\right]$$

For the third-order form:

$$M_1 = G_0$$

$$M_2 = 5M_1 - 3K_{S,0}G'_0$$

$$L_1 = K_{S,0} + \frac{4}{3}M_1$$

$$L_2 = 5L_1 - 3K_{S,0}\left(K'_{S,0} + \frac{4}{3}G'_0\right)$$

$$C_1 = 3L_1 - 4M_1$$

$$C_2 = 3L_2 - 4M_2 + 7C_1$$

For the fourth-order form:

$$M_3 = 9G''_0K_{S,0}^2 - 3(K'_{S,0} - 4)M_2 + 5(3K'_{S,0} - 5)M_1$$

$$L_3 = 9K''_{S,0}K_{S,0}^2 + 12G''_0K_{S,0}^2 - 3(K'_{S,0} - 4)L_2 + 5(3K'_{S,0} - 5)L_1$$

$$C_3 = 3L_3 - 4M_3 + 9C_2$$

For each pressure, the finite-strain equation is solved numerically for ε. Ringwoodite is calculated with the fourth-order formulation, whereas garnet-majorite is calculated with the third-order formulation. Thermoelastic parameters for the residual solid phases were taken following the compilation and modelling approach of Zhou et al.,[16] with individual endmember properties derived from published mineral-physics constraints. For ringwoodite, the forsterite endmember was parameterized using [17–20], whereas the fayalite endmember used [20–22]. Garnet-majorite endmembers were parameterized using pyrope, almandine, grossular, Mg-majorite and Fe-majorite thermoelastic properties. Pyrope was based on [19,23–26]. Almandine was based on [19,27] and grossular on [19,28]. Mg-majorite used [19,23–26,29]. Fe-majorite was based on [19,23,30,31].

Endmember thermoelastic parameters used in the Monte Carlo model: density and bulk/shear modulus terms.

Endmember	ρ_0 (g cm⁻³)	K_S (GPa)	G (GPa)	∂K_S/∂P	∂²K_S/∂P² (GPa⁻¹)	∂K_S/∂T (GPa K⁻¹)
Fo_ring	3.569	187.0 ± 1.0	121.1 ± 0.5	3.8 ± 0.1	0.0	−0.014 ± 0.003
Fa_ring	4.848	204.0 ± 5.0	103.0 ± 5.0	4.0	0.0	−0.014 ± 0.003
Py	3.560 ± 0.020	171.0 ± 0.5	94.9 ± 0.2	4.4 ± 0.1	0.0	−0.014 ± 0.003
Alm	4.319 ± 0.002	174.2 ± 1.2	94.9 ± 0.7	4.61 ± 0.14	0.0	−0.0267 ± 0.0007
Gr	3.605 ± 0.002	171.2 ± 0.8	107.4 ± 0.2	4.47 ± 0.02	0.0	−0.0138 ± 0.0003
Mg_Maj	3.560 ± 0.002	162.0 ± 0.5	86.2 ± 0.2	4.4 ± 0.1	0.0	−0.014 ± 0.003
Fe_Maj	4.409	180.24	88.7 ± 0.7	4.2 ± 0.2	0.0	−0.014 ± 0.003

Endmember thermoelastic parameters used in the Monte Carlo model: shear derivatives and thermal-expansion terms.

Endmember	∂G/∂P	∂²G/∂P² (GPa⁻¹)	∂G/∂T (GPa K⁻¹)	a_0	a_1	a_2
Fo_ring	1.5 ± 0.1	−0.046 ± 0.014	−0.012 ± 0.002	0.2497	0.3639	−0.6531
Fa_ring	1.5 ± 0.1	−0.046 ± 0.014	−0.012 ± 0.002	0.2697	0.0	0.0
Py	1.15 ± 0.06	0.0	−0.011 ± 0.002	0.2880	0.2787	−0.5521
Alm	1.06 ± 0.06	0.0	−0.0131 ± 0.0008	0.2600 ± 0.0500	2.3000 ± 1.4000	0.0
Gr	1.29 ± 0.05	0.0	−0.0128 ± 0.0002	0.1951	0.8089	−0.4972
Mg_Maj	1.15 ± 0.06	0.0	−0.011 ± 0.002	0.2880	0.2787	−0.5521
Fe_Maj	1.28 ± 0.08	0.0	−0.011 ± 0.002	0.2880	0.2787	−0.5521

Ringwoodite-majorite mass balance

The proportions of ringwoodite and garnet-majorite in the aggregate are calculated by least-squares mass balance using the starting composition and the measured phase compositions:

$$\min_{\mathbf{w}} \|\mathbf{A}\mathbf{w} - \mathbf{b}\|^2$$

subject to:

$$w_i \geq 0$$

$$\sum_i w_i = 1$$

where **w** contains the weight fractions of ringwoodite and garnet-majorite, **A** contains the phase oxide compositions, and **b** is the starting composition.

The oxides included in the mass balance are:

$$\mathrm{SiO_2},\ \mathrm{Al_2O_3},\ \mathrm{FeO},\ \mathrm{MgO},\ \mathrm{CaO}$$

Na_2O is not included in the mass balance because it is absent from the analysed ringwoodite and garnet-majorite compositions.

The residual is:

$$\mathbf{r}_{mb} = \mathbf{A}\mathbf{w} - \mathbf{b}$$

The calculated weight fractions are converted to volume fractions at each pressure using the phase densities:

$$\phi_i(P,T) = \frac{w_i/\rho_i(P,T)}{\sum_j w_j\,/\rho_j(P,T)}$$

Aggregate density

The density of the aggregate is calculated from the phase volume fractions:

$$\rho_{\mathrm{agg}}(P,T) = \sum_i \phi_i\,(P,T)\rho_i(P,T)$$

The aggregate elastic moduli are calculated using Voigt-Reuss-Hill averaging. The Voigt bounds are:

$$K_V = \sum_i \phi_i\,K_i$$

$$G_V = \sum_i \phi_i \, G_i$$

The Reuss bounds are:

$$K_R = \left(\sum_i \frac{\phi_i}{K_i} \right)^{-1}$$

$$G_R = \left(\sum_i \frac{\phi_i}{G_i} \right)^{-1}$$

The Voigt-Reuss-Hill averages are:

$$K_{\mathrm{VRH}} = \frac{K_V + K_R}{2}$$

$$G_{\mathrm{VRH}} = \frac{G_V + G_R}{2}$$

Monte Carlo uncertainty propagation

Uncertainties in the thermoelastic parameters are propagated using Monte Carlo sampling. For each endmember and each parameter with reported uncertainty, the parameter is sampled from a normal distribution:

$$p_i^{(k)} \sim \mathcal{N}(p_i, \sigma_i)$$

where $p_i$is the nominal parameter value and σ_i is its uncertainty.

Parameters without reported uncertainty are kept fixed. For each Monte Carlo realization, the full calculation is repeated from endmember sampling to aggregate VRH averaging.

The mean and standard deviation are calculated as:

$$\bar{x}(P) = \frac{1}{N} \sum_{k=1}^{N} x_k \, (P)$$

$$\sigma_x(P) = \sqrt{\frac{1}{N-1} \sum_{k=1}^{N} [x_k(P) - \bar{x}(P)]^2}$$

The 16th and 84th percentiles are used as an approximate 1σ interval, and the 2.5th and 97.5th percentiles are used as an approximate 95% interval. The nominal number of Monte Carlo realizations is $N = 500$.

Model assumptions and limitations

The model makes the following assumptions:

1. Ringwoodite is represented as an ideal binary Fo-Fa solid solution.
2. Garnet-majorite is represented by a five-endmember least-squares projection.
3. Endmember thermoelastic parameters are assumed applicable to the projected solid solutions.
4. The nominal solid-solution molar volumes are ideal.
5. Non-ideal volume effects are explored only through a simple excess-volume sensitivity test.
6. Elastic moduli of solid solutions are mixed linearly at the endmember level.
7. The aggregate elastic properties are calculated using Voigt-Reuss-Hill averaging.
8. The phase proportions are calculated from a two-phase mass balance using ringwoodite and garnet-majorite compositions.
9. Na_2O is excluded from the mass balance because it is not represented in the measured solid phases.
10. The model does not include additional phases, textural effects, anisotropy, grain-scale elastic interactions, or compositional zoning.

The excess-volume test is intended only as a sensitivity analysis. It does not imply that the true excess molar volume is known. Positive and negative values are tested because non-ideal solid solutions can display either expansion or contraction relative to ideal mixing depending on the endmember pair and crystal structure.

References

1. Agee, C. B. & Draper, D. S. Experimental constraints on the origin of Martian meteorites and the composition of the Martian mantle. *Earth Planet. Sci. Lett.* **224**, 415–429 (2004).
2. Duncan, M. S., Schmerr, N. C., Bertka, C. M. & Fei, Y. Extending the Solidus for a Model Iron-Rich Martian Mantle Composition to 25 GPa. *Geophys. Res. Lett.* **45**, 10,211-10,220 (2018).
3. Bertka, C. M. & Fei, Y. Mineralogy of the Martian interior up to core-mantle boundary pressures. *J. Geophys. Res. Solid Earth* **102**, 5251–5264 (1997).
4. Matsukage, K. N., Nagayo, Y., Whitaker, M. L., Takahashi, E. & Kawasaki, T. Melting of the Martian mantle from 1.0 to 4.5 GPa. *Journal of Mineralogical and Petrological Sciences* **108**, 201–214 (2013).
5. Collinet, M., Médard, E., Charlier, B., Vander Auwera, J. & Grove, T. L. Melting of the primitive martian mantle at 0.5-2.2 GPa and the origin of basalts and alkaline rocks on Mars. *Earth Planet. Sci. Lett.* **427**, 83–94 (2015).
6. Herzberg, C., Gasparik, T. & Sawamoto, H. Origin of mantle peridotite - constraints from melting experiments to 16.5 GPa. *Journal of Geophysical Research-Solid Earth and Planets* **95**, 15779–15803 (1990).
7. Agee, C. B., Li, J., Shannon, M. C. & Circone, S. Pressure-temperature phase-diagram for the Allende meteorite. *Journal of Geophysical Research-Solid Earth* **100**, 17725–17740 (1995).
8. Pierru, R. *et al.* Melting properties and phase relations of the Martian mantle from in situ measurements on iron-rich mineral assemblages. *Physics of the Earth and Planetary Interiors* **in review**, (2026).
9. Samuel, H. *et al.* Geophysical evidence for an enriched molten silicate layer above Mars's core. *Nature* **622**, 712–717 (2023).
10. Khan, A. *et al.* Evidence for a liquid silicate layer atop the Martian core. *Nature* **622**, 718–723 (2023).
11. Hernlund, J., Leinenweber, K., Locke, D. & Tyburczy, J. A. A numerical model for steady-state temperature distributions in solid-medium high-pressure cell assemblies. *American Mineralogist* **91**, 295–305 (2006).

12. Asimow, P. D. Melts under extreme conditions from shock experiments. in *Magmas Under Pressure: Advances in High-Pressure Experiments on Structure and Properties of Melts* 387–418 (Elsevier, 2018). doi:10.1016/B978-0-12-811301-1.00015-0.
13. Wakabayashi, D. & Funamori, N. Equation of state of silicate melts with densified intermediate-range order at the pressure condition of the Earth's deep upper mantle. *Phys. Chem. Miner.* **40**, 299–307 (2013).
14. Jing, Z. & Karato, S. ichiro. A new approach to the equation of state of silicate melts: An application of the theory of hard sphere mixtures. *Geochim. Cosmochim. Acta* **75**, 6780–6802 (2011).
15. Kono, Y. *et al.* Gravitational stability of iron-rich peridotite melt at Mars' core-mantle boundary. *Commun. Earth Environ.* **6**, 148 (2025).
16. Zhou, W.-Y. *et al.* High pressure-temperature phase equilibrium studies on Martian basalts: Implications for the failure of plate tectonics on Mars. *Earth Planet. Sci. Lett.* **594**, 117751 (2022).
17. Suzuki, i., ohtani, e. & kumazawa, m. Thermal expansion of ringwoodite mg2sio4 *Journal of Physics of the Earth* **27**, 53–61 (1979).
18. Higo, Y., Inoue, T., Irifune, T., Funakoshi, K. & Li, B. Elastic wave velocities of (Mg0.91Fe0.09)2SiO4 ringwoodite under P–T conditions of the mantle transition region. *Physics of the Earth and Planetary Interiors* **166**, 167–174 (2008).
19. Fei, Y. Thermal Expansion. in *Mineral Physics & Crystallography* 29–44 (1995). doi:https://doi.org/10.1029/RF002p0029.
20. Schulze, K. *et al.* Seismically invisible water in Earth's transition zone? *Earth Planet. Sci. Lett.* **498**, 9–16 (2018).
21. Armentrout, M. & Kavner, A. High pressure, high temperature equation of state for Fe2SiO4 ringwoodite and implications for the Earth's transition zone. *Geophys. Res. Lett.* **38**, (2011).
22. Yamanaka, T. Crystal structures of Ni2SiO4 and Fe2SiO4 as a function of temperature and heating duration. *Phys. Chem. Miner.* **13**, 227–232 (1986).
23. Suzuki, i. & anderson, o. L. Elasticity and thermal expansion of a natural garnet up to 1, 000k. *Journal of Physics of the Earth* **31**, 125–138 (1983).
24. Irifune, T. *et al.* Sound velocities of majorite garnet and the composition of the mantle transition region. *Nature* **451**, 814–817 (2008).

25. Liu, J., Chen, G., Gwanmesia, G. D. & Liebermann, R. C. Elastic wave velocities of pyrope–majorite garnets (Py62Mj38 and Py50Mj50) to 9 GPa. *Physics of the Earth and Planetary Interiors* **120**, 153–163 (2000).
26. Sinogeikin, S. V & Bass, J. D. Elasticity of Majorite and a Majorite-Pyrope solid solution to high pressure: Implications for the Transition Zone. *Geophys. Res. Lett.* **29**, 4-1-4–4 (2002).
27. Arimoto, T., Gréaux, S., Irifune, T., Zhou, C. & Higo, Y. Sound velocities of Fe3Al2Si3O12 almandine up to 19GPa and 1700K. *Physics of the Earth and Planetary Interiors* **246**, 1–8 (2015).
28. Gwanmesia, G. D., Wang, L., Heady, A. & Liebermann, R. C. Elasticity and sound velocities of polycrystalline grossular garnet (Ca3Al2Si3O12) at simultaneous high pressures and high temperatures. *Physics of the Earth and Planetary Interiors* **228**, 80–87 (2014).
29. Hao, M. *et al.* The seismically fastest chemical heterogeneity in the Earth's deep upper mantle—implications from the single-crystal thermoelastic properties of jadeite. *Earth Planet. Sci. Lett.* **543**, 116345 (2020).
30. Ismailova, L. *et al.* Effect of composition on compressibility of skiagite-Fe-majorite garnet. *American Mineralogist* **102**, 184–191 (2017).
31. Murakami, M., Sinogeikin, S. V, Litasov, K., Ohtani, E. & Bass, J. D. Single-crystal elasticity of iron-bearing majorite to 26 GPa: Implications for seismic velocity structure of the mantle transition zone. *Earth Planet. Sci. Lett.* **274**, 339–345 (2008).
